# Understanding tumor adaptations and resistance to MET inhibitors in MET-altered non-small cell lung cancer

**DOI:** 10.1016/j.neo.2026.101359

**Published:** 2026-09-14

**Authors:** Manon A. Simard, Elba Marín, Silvia García-Roman, Cristina Aguado, Michail Yekelchyk, Carolin Schmelas, Nina Berges, Balca R. Mardin, Sophie Ockfen, Rebecca Schaefer, Markus Esser, Jing Yang, Felix Geist, Oliver Schadt, Christof Reusch, Sebastian Bender, Núria Jordana-Ariza, Carlos Esparré, Ruth Román, Tolulope Apata, Cristina Teixido, Marc Rico-Pastó, Sara Hijazo-Pechero, Marina Querol, Marie Morfouace, Miguel Ángel Molina-Vila, Noemí Reguart, Niki Karachaliou

**Affiliations:** a Research Unit Oncology, the healthcare business of Merck KGaA, Darmstadt, Germany; b Hospital Clinic, Barcelona, Spain; c Department of Medicine, University of Barcelona, Barcelona, Spain; d Pangaea Oncology, Hospital Universitari Dexeus, Barcelona, Spain; e Lab Services, Site Management, the healthcare business of Merck KGaA, Darmstadt, Germany; f Discovery Development Technologies, the healthcare business of Merck KGaA, Darmstadt, Germany; g Facultat de Medicina i Ciències de la Salut, Universitat de Barcelona, Barcelona, Spain; h Translational Genomics and Targeted Therapeutics in Solid Tumors, IDIBAPS, Barcelona, Spain; i Molecular Biology CORE & Department of Pathology, Hospital Clinic Barcelona, Barcelona, Spain; j Institute for Bioengineering of Catalonia (IBEC), The Barcelona Institute for Science and Technology (BIST), Barcelona, Spain; k Unit of Biophysics and Bioengineering, Department of Biomedicine, School of Medicine and Health Sciences, Universitat de Barcelona, Barcelona, Spain; l Global Clinical Development, Oncology, the healthcare business of Merck KGaA, Darmstadt, Germany

**Keywords:** Lung cancer (NSCLC), MET exon 14, MET inhibition, Resistance, Spatial transcriptomics, CRISPR screen

## Abstract

**Aim:**

Type Ib MET inhibitors are clinically active in selected MET-altered non-small cell lung cancer, particularly tumors with *MET* exon 14 skipping or *MET* amplification, but acquired resistance remains incompletely understood. Here, we investigated resistance across biologically distinct MET-altered contexts, including *MET* exon 14 skipping, *MET* amplification, and MET overexpression.

**Methods:**

Paired baseline and progression samples from seven patients treated with tepotinib or capmatinib were analyzed using spatial transcriptomics, whole-exome sequencing, RNA sequencing, CRISPR screening, and drug-combination assays. Patient-derived cultures and resistant cell-line models were used to explore resistance-associated changes.

**Results:**

MET inhibitor resistance was heterogeneous, with persistence of the initial MET alteration in most evaluable cases and emergence of patient-specific genomic events. Three main resistance-associated, often overlapping, routes were identified: on-target MET evolution through kinase-domain alterations; extracellular matrix and tumor-microenvironment remodeling, including collagen and fibronectin upregulation, complement-related signaling, and partial EMT-associated programs; and bypass signaling involving EGFR/HER, MAPK, and PI3K/Akt pathways. *In vitro* models reproduced several tumor-cell-intrinsic features but only partially captured microenvironment-associated changes.

**Conclusions:**

MET inhibitor resistance in this cohort involved overlapping, context-dependent genomic, phenotypic, and signaling adaptations, supporting combination strategies for MET-altered lung cancer.

## Highlights

• MET inhibitor resistance is heterogeneous across distinct MET-altered NSCLC.
• Spatial transcriptomics reveal shared ECM remodeling in tumors upon progression.
• *In vitro* models partially recapitulate aspects of clinical MET inhibitor resistance.
• CRISPR and combination drug screens reveal overlapping functional dependencies.
• Dual targeting of EGFR/MAPK/PI3K with MET restores sensitivity in resistance settings.

## Introduction

Aberrant signaling of the hepatocyte growth factor receptor (MET) receptor tyrosine kinase has emerged as a driver of oncogenesis [1]. In non-small cell lung cancer (NSCLC), primary MET alterations include *MET* exon 14 skipping (*MET*ex14; 3–4 %) [1,2], *MET* amplification (*MET*amp; 1–6 %) [3,4], MET protein overexpression (MET-OE; 20–25 %) [5,6], and *MET* fusions (0.2–0.3 %) [7]. Secondary *MET*amp and MET-OE are the most frequent bypass mechanisms among patients who develop acquired resistance to EGFR-targeted therapies [[8], [9], [10]]. Furthermore, *MET*ex14 skipping and *MET*amp co-occur in 7.6–13.8 % of cases [11,12], underscoring that MET-altered NSCLC comprises biologically related but heterogeneous disease subsets with potentially different degrees of MET dependency. Herein, MET-altered NSCLC refers to both *MET* genetic alterations (*MET*ex14 skipping or *MET*amp) as well as MET-OE.

Several MET tyrosine kinase inhibitors (TKIs) have entered clinical use in MET-altered NSCLC. These agents can be broadly classified into three types based on their interaction with the adenosine triphosphate (ATP) pocket [13]. Type I TKIs competitively target the active conformation of MET and are subdivided into Type Ia (crizotinib, ensartinib) and Type Ib (tepotinib, capmatinib, savolitinib, gumarontinib, bozitinib). Type Ib inhibitors exhibit higher MET selectivity, translating into potent clinical activity in tumors harboring *MET*ex14 skipping or *MET*amp [[14], [15], [16]]. Tepotinib was the first oral MET inhibitor (METi) approved in Japan for patients with unresectable, advanced, or recurrent NSCLC carrying *MET*ex14 skipping [14], and subsequently received approvals in additional regions. Capmatinib [16] and savolitinib [17] have also been approved for *MET*ex14-skipping NSCLC. Type II TKIs (cabozantinib, merestinib, glesatinib, foretinib) also bind to the ATP site but stabilize the inactive kinase conformation [18,19], resulting in broader off-target effects [20]. Type III drugs, such as tivantinib, bind allosterically within a pocket distinct from the ATP‐binding site [21].

Despite promising response rates, drug resistance commonly arises, although the underlying mechanism frequently remains unknown. Known resistance mechanisms include on-target *MET* kinase domain mutations (e.g., D1228 or Y1230) and activation of bypass pathways such as Kirsten rat sarcoma viral oncogene homolog-mitogen-activated protein kinase (KRAS-MAPK) or epidermal growth factor receptor-human epidermal growth factor receptor (EGFR-HER), which circumvent MET blockade [22]. In the VISION study, comprehensive liquid and tissue biopsy analyses from tepotinib‐treated patients with *MET*ex14 skipping NSCLC revealed the complex interplay of on‐target *MET* kinase domain mutations, bypass pathway alterations (EGFR, KRAS), and negative prognostic factors (*TP53* mutations) [23]. However, most prior studies have primarily focused on genomic alterations in small cohorts or individual models [11,12,24,25]. Consequently, the extent to which diverse resistance events converge on shared biological programs across *MET*ex14 skipping, *MET*amp, and MET-OE remains incompletely defined. This gap is particularly relevant because post-progression tissue specimens are scarce, limiting the ability to distinguish subgroup-specific resistance mechanisms from broader resistant-state adaptations.

In this context, preclinical models enable hypothesis-driven investigations to dissect METi resistance. Conventional tumor-derived cell lines can be treated and monitored over time, providing an adaptable system to model emergent resistance [26]. However, such models cannot fully reproduce the tumor microenvironment (TME), stromal context, immune architecture, and spatial organization of resistant tumors. Multi-omics approaches, including spatial transcriptomics, whole exome sequencing (WES), RNA sequencing, CRISPR-based functional genomics screens, and synergy assessment of targeted therapy combinations, can leverage clinically derived materials and well-characterized *in vitro* models to distinguish recurrent resistance programs from model- or patient-specific findings.

In this study, we investigated paired baseline and post-progression biopsies and patient-derived primary cultures from patients with MET-altered NSCLC treated with Type Ib METi. Clinical findings were complemented by *in vitro* models that developed resistance to METi after prolonged exposure. By integrating large-scale genomic analyses, transcriptomic profiling (RNA sequencing and spatial transcriptomics), CRISPR-based functional screening, synergy assays, and Western blot, we sought to define not only individual resistance events but also the broader biological framework linking genomic evolution, extracellular matrix (ECM) remodeling, immune/microenvironmental features, and bypass signaling. This integrated approach is intended to clarify which aspects of METi resistance are shared across heterogeneous MET-altered settings, which remain context dependent, and which may support rational combination strategies.

## Methods

### Patients

Patients with advanced non-squamous NSCLC with MET alterations were retrospectively included following acquired resistance to Type Ib METi (tepotinib or capmatinib). Inclusion was limited to patients with clinical samples and/or molecular information available both before treatment initiation and after progression on METi therapy (n = 7). All patients provided informed consent, and the study was approved by the Local Ethical Committee (Comité Ético de Investigación Clínica del Hospital Clínic de Barcelona or Grupo Quirón Salud) and conducted in accordance with the principles of the Declaration of Helsinki.

Information collected included sex, age, histology, TNM stage at diagnosis (seventh edition of the American Joint Committee on Cancer), smoking history, molecular characteristics at baseline and progression, previous treatments, and clinical outcomes.

### Immunohistochemistry (IHC)

IHC staining for total and phospho-MET was performed with MET SP44 clone (Ventana, Roche) and MET phospho-Y1234/1235 (D26; Cell Signaling, 3077) on a BenchMark ULTRA automated tissue staining system (Ventana Medical Systems, Tucson, USA). Samples were considered positive when intense membrane staining (3+) was observed in >50 % of tumor cells, upon pathologist assessment.

### Fluorescent *in situ* hybridization (FISH)

FISH for *MET* was performed with the ZytoLight SPEC *MET/CEP7* Dual Color Probe (ZytoVision, Z-2087). A sample was considered positive for *MET*amp if (i) a *MET/CEP7* ratio >2 and (ii) a *MET* gene copy number >6 per cell were observed.

### Primary culture generation

Three primary cultures derived from NSCLC patient malignant pleural effusions (5-5000 mL) were generated through isolating cells by centrifugation, erythrocytes removal, and culturing the remaining cells in Roswell Park Memorial Institute medium (RPMI)-1640 (Gibco, 11875093), supplemented with 10 % human serum (Sigma-Aldrich, H4522), 50 µg/mL penicillin-streptomycin (Gibco, 10378016) and 2 mM L-glutamine (Gibco, 25030081; [[27], [28], [29]]). Primary cultures were cultured in a humidified atmosphere with 5 % CO_2_ at 37 °C, assessed weekly for mycoplasma (Venor®GeM Classic kit, Minerva Biolabs, 11-1025), and authenticated by monthly genotyping for driver alterations. After 15 passages, cells were discarded and a low passage vial was thawed.

### Spatial transcriptomics

GeoMx DSP (NanoString, Bruker Spatial) was performed on 5 µm thick formalin-fixed paraffin-embedded (FFPE) sections within a week of microtome sectioning to ensure sufficient RNA quality. An adjacent section was subjected to hematoxylin and eosin (H & E) staining using a Ventana Symphony H & E autostainer (Roche) to inform ROI selection. GeoMx DSP was performed as previously described [30]. Further details on the protocol and analysis methods used can be found in the supplementary material.

### Commercial cell lines

The EBC-1 cell line was purchased from HSRRB and cultured in Minimum Essential Medium (MEM; Sigma, M2279) with 10 % fetal bovine serum (FBS; Millipore, S0615) and 1 % GlutaMAX™ supplement (Gibco, 35050061). The Hs746T cell line was purchased from ATCC and cultured either in RPMI-1640 medium (Gibco, 11875093) or Dulbecco’s Modified Eagle’s Medium (DMEM; Gibco, 11965092) with 10 % FBS, 1 mM sodium-pyruvate (Gibco, 11306670) and 1 % GlutaMAX™ supplement. The MKN-45 cell line was purchased from DSMZ and cultured in RPMI-1640 with 20 % FBS and 1 % GlutaMAX™ supplement. Cultures were maintained at 37°C in a humidified 5 % CO_2_ atmosphere. Mycoplasma testing was routinely performed using the Venor®GeM Classic kit (Minerva Biolabs, 11-1025).

### In-house generation of METi-resistant cell lines

Tepotinib-resistant EBC-1 (two clones; TR2 and TR3), MKN-45, and Hs746T cells were generated as previously described [31]. Additionally, Hs746T cells resistant to other METi were established through continuous exposure to progressively higher concentrations of crizotinib, tepotinib, capmatinib, or cabozantinib for a minimum duration of 6 months. Four resistant cell models were established: Hs746T-CriR (crizotinib-resistant), Hs746T-TR2 (tepotinib-resistant), Hs746T-CapR (capmatinib-resistant), and Hs746T-CabR (cabozantinib-resistant).

### DNA purification and next-generation sequencing (NGS)

DNA extraction from tissue and liquid biopsies, primary cultures, and cell models was conducted using the DNeasy® Blood & Tissue Kit (Qiagen, 69504), DNA concentration was measured by Qubit® and/or NanoDrop™ One (Thermo Fisher Scientific) and quality was assessed by gel agarose electrophoresis and qPCR. Purity index A260/A230 was acceptable when situated between 1.8 and 2.2. Sequencing panel and further NGS experimental and analysis details can be found in the Supplementary Methods.

### RNA purification and nCounter

RNA extraction from generated METi-resistant cells was performed using the High Pure RNA Isolation Kit (Roche, 11828665001). Concentrations were estimated using the Qubit® 4.0 Fluorometer-High Sensitivity (Thermo Fisher Scientific). For quality analysis, a 4200 TapeStation System (Agilent Technologies) was used.

For the nCounter assay (NanoString, Bruker Spatial), total RNA was directly hybridized with customized code sets designed to detect specific fusion transcripts (*ALK, ROS1, RET, NTRK1/2/3, NRG1, FGFR1/2/3*), splicing variants (*MET*ex14, *EGFR*vIII) and gene expression (*ALK, ROS1, RET, MET, NTRK1, NTRK2, NTRK3, NRG1, FGFR1, FGFR2, FGFR3, CD274, CD4, CD8A, FOXP3, GZMM, IFNG, PDCD1)*. Hybridization steps were performed in a Verity thermal cycler (Applied Biosystems). All processes of capture, clean-up, and digital data acquisition were performed with nCounter Prep Station™ and Digital Analyzer™ (NanoString, Bruker Spatial). The number of counts for each gene was extracted from the nCounter-generated RCC files using nSolver analysis Software (v4.0.70). RNA counts were normalized using positive controls and housekeeping transcripts, as previously described [32].

### RNA sequencing

Bulk RNA sequencing of the Hs746T-derived METi-resistant cell lines (cabozantinib, capmatinib, crizotinib, tepotinib, and parental control), was performed on a NovaSeq 6000 platform (Illumina, USA), using a 2 × 150 bp paired-end read configuration and an average of 50 million reads per sample. Quality control analyses were performed prior to RNA sequencing. Samples were quantified by Qubit® and run by capillary electrophoresis to check their integrity.

For single-cell RNA sequencing of tepotinib-resistant cell lines (EBC-1, Hs746T, MKN-45, with parental control counterparts), cell suspensions were prepared for processing with the 10X Chromium Next GEM Cell 3′ kit (PN-1000121), for the GEM generation and barcoding, clean-up, cDNA amplification, and gene expression library construction, according to manufacturer’s instructions. Resulting libraries were quantified by Qubit® and integrity was assessed on the 2100 Bioanalyzer (Agilent Technologies) with the DNA 1000 kit (5067-1504). Approximately 8,000 cells with >50,000 reads per cell (sample reads between 400 and 600 million) were sequenced using the NovaSeq 6000 platform using an S2 flow cell with 2 × 100 bp reads at IMGM Laboratories (Martinsried, Germany). A detailed description of the RNA sequencing analysis is found in the Supplementary Methods.

### Whole exome sequencing (WES)

Patient samples were prepared for sequencing using the SureSelectXT Human All Exon (50 Mb) V5 kit (Agilent Technologies, 5190-6213). Libraries were synthesized and sequenced on the Illumina HiSeq2500 platform to generate at least 125 million, 2 × 100 bp paired end reads. Reads were aligned to the reference human genome (hg19) and BAM files were generated.

Cell line extracted DNA was prepared using either the DNA Prep with Exome 2.0 Plus Enrichment kit (Illumina, 20077596) or KAPA HyperExome (Roche, 09062548001). Libraries were synthesized and sequenced on an Illumina NextSeq2000 or NovaSeq6000 platform with 2 × 150 bp paired end reads and an average coverage of 150x. Detailed analysis methods and interpretation of results can be found in the Supplementary Methods.

### *In vitro* cell viability

Cells were seeded in 96-well plates in the corresponding growth medium. Twenty-four hours later, inhibitor monotherapy treatment was initiated for 72–96 h. Viability readouts were performed using either 0.75 ng/mL Thiazolyl Blue Tetrazolium Bromide (MTT; Sigma-Aldrich, 475989) for 1–2 h at 37°C or using CellTiter Glo (Promega, G7572). MTT readouts were performed by reading the absorbance at 565 nm, while CellTiter Glo readouts were performed by measuring the luminescence after 500 ms integration time, using an Infinite M Plex Tecan microplate reader (Mannedorf, Switzerland). A control condition without the drug but containing the maximum DMSO concentration was also included.

Data were derived from at least three independent experiments and presented as mean ± standard deviation. Dose–response curves and IC_50_ values were determined using non-linear regression analysis implemented in GraphPad Prism 10.0. A p-value lower than 0.05 was considered for statistical significance.

### *In vitro* synergy testing

Synergy testing was performed by combining tepotinib treatment with 46 compounds (compound details in Supplementary Table S1) in 384-well black, flat-bottom plates (Corning, 3571), using an 8 × 8 matrix using ½ log increments, and concentration range of 15 µM – 5 nM in most cases, except for alisertib, binimetinib, dactolisib, paclitaxel, everolimus, trametinib, AZD8055, ulixertinib, and apitolisib, since these inhibitors were too potent at such concentrations. A monotherapy condition for each inhibitor, along with a DMSO-only negative control and a 30 µM doxorubicin hydrochloride (Sigma-Aldrich, D1515) positive control, was also present on each plate. The D300e dispenser (Tecan, Mannedorf, Switzerland) was used to dispense all compounds, one day after seeding cells. After 96 h of combination treatment, a CellTiter Glo (Promega, G7572) readout was performed. To determine synergistic potential and scores, a Loewe synergy calculation was performed using GeneData Screener. Testing was repeated when it did not meet quality control criteria.

### CRISPR screen

The genome-wide lentiviral CRISPR KO library was used (details in the Supplementary Methods). Two cell lines, the low passage EBC-1 cells and one tepotinib-resistant EBC-1 clone were transduced with a lentiviral vector expressing doxycycline-inducible Cas9. The Cas9-EBC-1 cells were subjected to a tepotinib-resistance screen (treated with tepotinib at an IC_70_ of 3.63 nM for 3 days), while the Cas9-containing resistant clone was used in a tepotinib-sensitivity screen (treated with 50 nM tepotinib every 3 to 4 days for a total of three consecutive treatment rounds and reached IC_50_).

Further information about the Cas9 cell line generation and CRISPR screens including sequencing and quality checks can be found in the Supplementary Methods.

### Data availability

Additional data are available from the authors upon reasonable request. Any requests for data by qualified scientific and medical researchers for legitimate research purposes will be subject to the healthcare business of Merck KGaA, Darmstadt, Germany’s Data Sharing Policy (https://ror.org/04vpzfb52). All requests should be submitted in writing to the healthcare business of Merck KGaA, Darmstadt, Germany’s data sharing portal (https://www.emdgroup.com/en/research/our-approach-to-research-and-development/healthcare/clinical-trials/commitment-responsible-data-sharing.html). When the healthcare business of Merck KGaA, Darmstadt, Germany has a co-research, co-development, or co-marketing or co-promotion agreement, or when the product has been out-licensed, the responsibility for disclosure might be dependent on the agreement between parties. Under these circumstances, the healthcare business of Merck KGaA, Darmstadt, Germany will endeavor to gain agreement to share data in response to requests.

## Results

### Clinical resistance of MET-altered NSCLC is characterized by genomic heterogeneity

To investigate mechanisms associated with acquired METi resistance, we analyzed paired baseline and progression samples from seven patients with MET-altered NSCLC treated with tepotinib (n = 4) or capmatinib (n = 3). Longitudinal specimens included tumor tissue, blood, and pleural effusion samples collected before treatment initiation and at disease progression. Primary cultures from pleural effusions at progression on tepotinib were established from three patients (TEP-153, TEP-1513, TEP-1894). Clinical information is summarized in Table 1.

**Table 1:** Clinical characteristics from patients treated with METi.

ID	Age	Sex	Smoking status	Site of metastases	Prior therapies for advanced disease	MET alteration	initiation of METi	Discontinuation of METi	Best response	Sample at baseline	Sample at progression	Primary culture at progression
TEP-153	62	M	Former smoker	N/A	1 cycle of cis/gem	*MET*amp	Jun 2021	Oct 2021	PR	Blood	Pleural effusion	Yes
TEP-158	68	M	Former smoker	Mediastinal nodes, bone	None	*MET*amp	Mar 2021	Dec 2021	PR	Tissue (lung)	Tissue (bone) and blood	No
TEP-1513	74	M	Former smoker	N/A	None	MET-OE	May 2021	Apr 2022	PR	Tissue (lung)	Tissue (lung) and pleural effusion	Yes
TEP-1894	66	M	Smoker	N/A	Carbo/pem+pembro	*MET*ex14 skipping	Nov 2022	May 2023	PR	Tissue (lung)	Pleural effusion	Yes
CAP-2	68	F	Non-smoker	Breast	Carbo/pem	*MET*ex14 skipping	Feb 2019	Aug 2019	PR	Tissue (lung)	Tissue (lung)	No
CAP-3	61	M	Former smoker	Bone, peritoneal implant	None	*MET*ex14 skipping	Nov 2019	Jul 2021	PR	Tissue (lung) and blood	Tissue (lung) and blood	No
CAP-4	72	M	Former smoker	Brain	None	*MET*ex14 skipping	Jul 2021	Mar 2022	PR	Tissue (lung) and blood	Tissue (lung) and blood	No

cis: cisplatin, gem: gemcitabine, carbo: carboplatin, pem: pemetrexed, pembro: pembrolizumab, PR: partial response, N/A: not applicable.

**Fig. 1:**
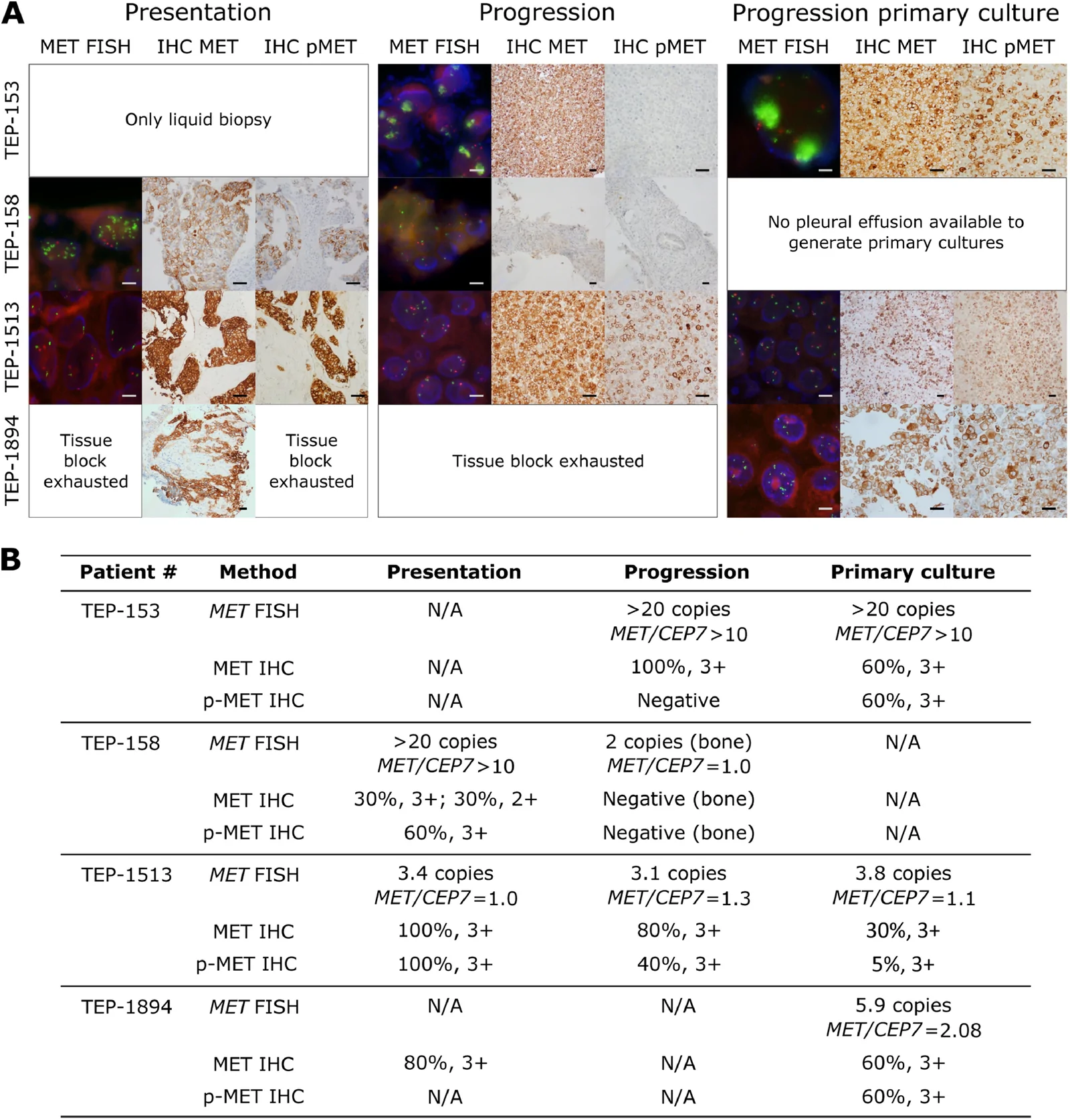
***MET* amplifications and MET overexpression in patient samples and primary cultures derived at progression to tepotinib. A**, *MET* FISH was performed to identify *MET* amplification (*MET* = green; *CEP7* = red, nuclei = blue) in patient samples either before and at disease progression to tepotinib treatment, or in primary cultures derived from pleural effusion at disease progression. In the same samples, IHC of MET protein expression and its phosphorylation were assessed, shown as brown staining. Error bars: grey (FISH) = 20 µm, black (IHC) = 100 µm. **B**, *MET* amplification and MET-overexpression quantitation of FISH and IHC of patient-derived samples. N/A: not applicable. For TEP-153 presentation, only blood was available. For TEP-158, no primary culture was established. For TEP-1894, tissue at presentation was exhausted prior to FISH and pMET IHC.

Genomic analyses confirmed substantial inter-patient heterogeneity both at baseline and upon disease progression (Supplementary Table S2). Baseline MET alterations included *MET*amp (n = 3) assessed by FISH or next-generation sequencing (NGS), MET-OE (n = 2) detected by nCounter RNA expression profiling, or *MET*ex14 skipping (n = 4) detected by nCounter or NGS (Fig. 1). Baseline co-alterations were identified in five patients and included mutations in *TP53, STK11, FAT1, FGFR1,* and *ERBB2,* as well as *MYC* amplification and *BRAF* loss (Supplementary Table S2).

At progression to tepotinib, *MET*amp or MET-OE persisted in all evaluable cases except TEP-158 (FISH or IHC). In this patient, *MET*amp was detected at baseline (FISH, nCounter, and NGS), but was no longer detected in the bone metastasis progression biopsy (FISH, IHC, or nCounter), while remaining detectable in blood- and tissue-based NGS. Progression samples and primary cultures displayed high concordance across detection methods, with one exception: TEP-1894, in which *KRAS* and *MET* amplifications were identified in the primary culture, whereas only *KRAS* amplification was detected in the corresponding pleural effusion by NGS.

No recurrent genomic resistance driver was identified across all progression samples. However, two patients acquired *MET* kinase domain alterations affecting residues D1228 and Y1230, consistent with established on-target resistance mechanisms, while individual patients acquired *ARID1A* or *MUTYH* mutations. Baseline co-alterations were generally conserved at progression, with *MYC* amplification and *ERBB2* mutation, representing the only notable exceptions. These findings underscore the complex molecular heterogeneity underlying METi resistance across *MET*ex14, *MET*amp, and MET-OE NSCLC.

### Spatial transcriptomics reveal shared biological programs upon METi resistance

To explore whether common biological programs emerge despite genomic heterogeneity, spatial transcriptomic profiling was performed on paired tissue-based biopsies from five patients: TEP-158 (*MET*amp) and TEP-1513 (MET-OE), progressing on tepotinib, and CAP-2, CAP-3, and CAP-4 (all *MET*ex14 skipping), progressing on capmatinib. Owing to differences in biopsy size and the presence of similar progression-associated transcriptional signatures across patients, samples were analyzed either collectively or stratified by METi treatment (tepotinib or capmatinib) to reduce noise and identify shared biological programs.

**Fig. 2:**
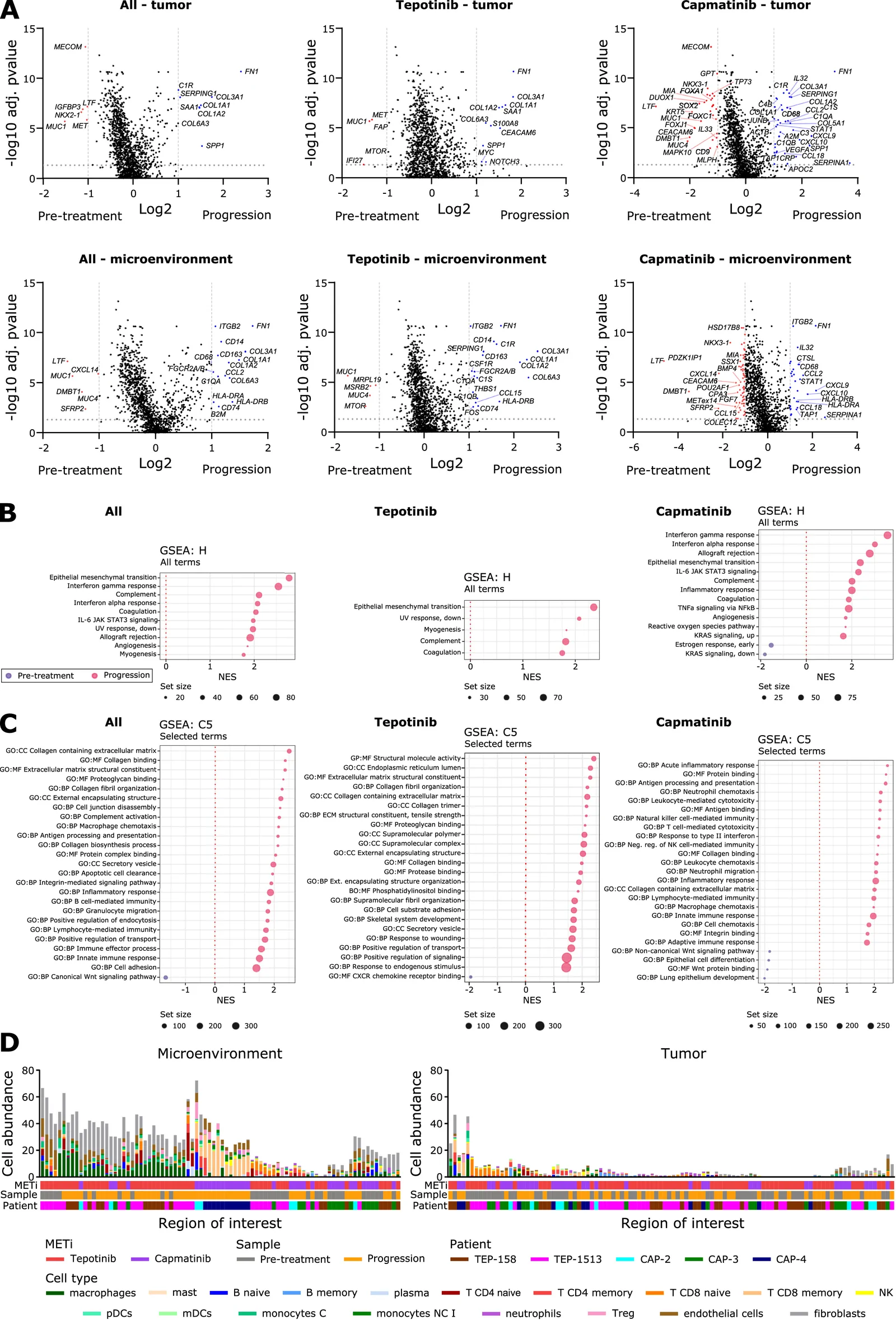
**Spatial transcriptomics of paired biopsies before and after progression to METi.** Whole transcriptome expression was determined in the tumor and microenvironment of patient samples before and after disease progression to tepotinib (n = 2 patients) or capmatinib (n = 3 patients). **A**, DEGs, represented by volcano plots with most significant DEGs annotated. Results from comparison of all pre-treatment samples and progression samples combined are shown, along with those for each METi, either in the tumor or the microenvironment compartment. Log fold change of 2 was used as DEG threshold with an adjusted p-value <0.05. Pre-treatment DEGs are in red, whereas progression DEGs are in blue. GSEA of C5 terms (gene ontology; **B**) and hallmarks (H; **C**) was performed to obtain an NES, represented as a dot plot for all samples together. For C5, only selected terms are shown as 245, 271, and 169 terms were output for the combined (all), tepotinib, and capmatinib patients, respectively. P-value cut-off used for H and C5 was p < 0.05. **D**, Abundance of immune and microenvironment cell types from each microenvironment and tumor region of all samples, obtained through deconvolution of region transcriptome. Regions are clustered based on cell type composition. METi, sample, and patient for each region can be seen below the graph.

Profiling of the tumor compartment and surrounding microenvironment was performed independently, based on PanCK staining, and identified consistent decreases in *MET, MUC1, NKX2-1, MECOM*, and the insulin-associated genes *IGFBP3* and *LTF* within the tumor compartment upon resistance (Fig. 2A). In contrast, the TME compartment was characterized by downregulation of *CXCL14, DMBT1, MUC4*, and *SFRP2,* together with upregulation of immune-related genes (*HLA-DRA, HLA-DRB, CD74, CD14, CD163, CD68, B2M*), suggesting the presence of tumor-associated macrophages and corroborated by cell-type deconvolution analyses (Fig. 2D). Across compartments, the upregulation of collagen genes (*COL1A1, COL1A2, COL3A1, COL6A3*) and fibronectin (*FN1*) were among the most prominent changes observed at progression, consistent with matrix remodeling and stromal adaptation. CD45 staining and cell-type deconvolution further demonstrated low lymphocyte density in both tumor and stromal regions at progression, accompanied by increased macrophage signatures.

Analyses restricted to either tepotinib- or capmatinib-treated patients alone, also reflecting different MET-alterations, produced comparable ECM, immune, and EMT-associated program findings. The main differences were the upregulation of *MYC, CEACAM6,* and *NOTCH3* in tepotinib-progressing tumors, compared with *STAT1* and *VEGFA* in capmatinib-progressing tumors. At baseline, the predominant differentially expressed genes were *VEGFA* and *HIF1A* in tumors later treated with tepotinib, and *TNC* in tumors later treated with capmatinib. Results varied more widely at the individual patient level, reflecting the limited tissue, thus lower total ROIs available, and the genomic differences observed between patients (Supplementary Figure S1).

Gene set enrichment analysis (GSEA) of differentially expressed genes (DEGs) corroborated these observations, identifying enrichment of pathways related to ECM organization, integrin signaling, and antigen presentation contributing to the stromal response at progression. Partial EMT-associated programs included modest enrichment of TGFβ signaling and transcriptional regulation pathways (Fig. 2B-C). KRAS signaling was enriched in two patients upon progression (one per METi), representing patient-specific bypass events within an otherwise shared ECM and immune remodeling landscape. Patient-level GSEA results are provided in Supplementary Figure S2.

Overall, spatial transcriptomic profiling revealed that although genomic resistance events remained heterogeneous, tumors and their microenvironments converged on shared ECM remodeling, immune adaptation, and partial EMT-associated programs upon acquired METi resistance.

### *In vitro* METi resistance models partially recapitulate patient resistant programs

To investigate resistance-associated biological programs in a controlled experimental setting, we established and characterized multiple METi-resistant *in vitro* models, including primary cultures derived from three tepotinib-progressing patient samples and resistant derivatives of MET-dependent cell lines: EBC-1 (*MET*amp, lung adenocarcinoma), Hs746T (*MET*ex14 skipping and *MET*amp, gastric cancer), and MKN-45 (*MET*amp, gastric cancer). These models enabled interrogation of biological adaptations associated with acquired resistance while providing a framework to compare patient-derived and experimental resistance states.

**Fig. 3:**
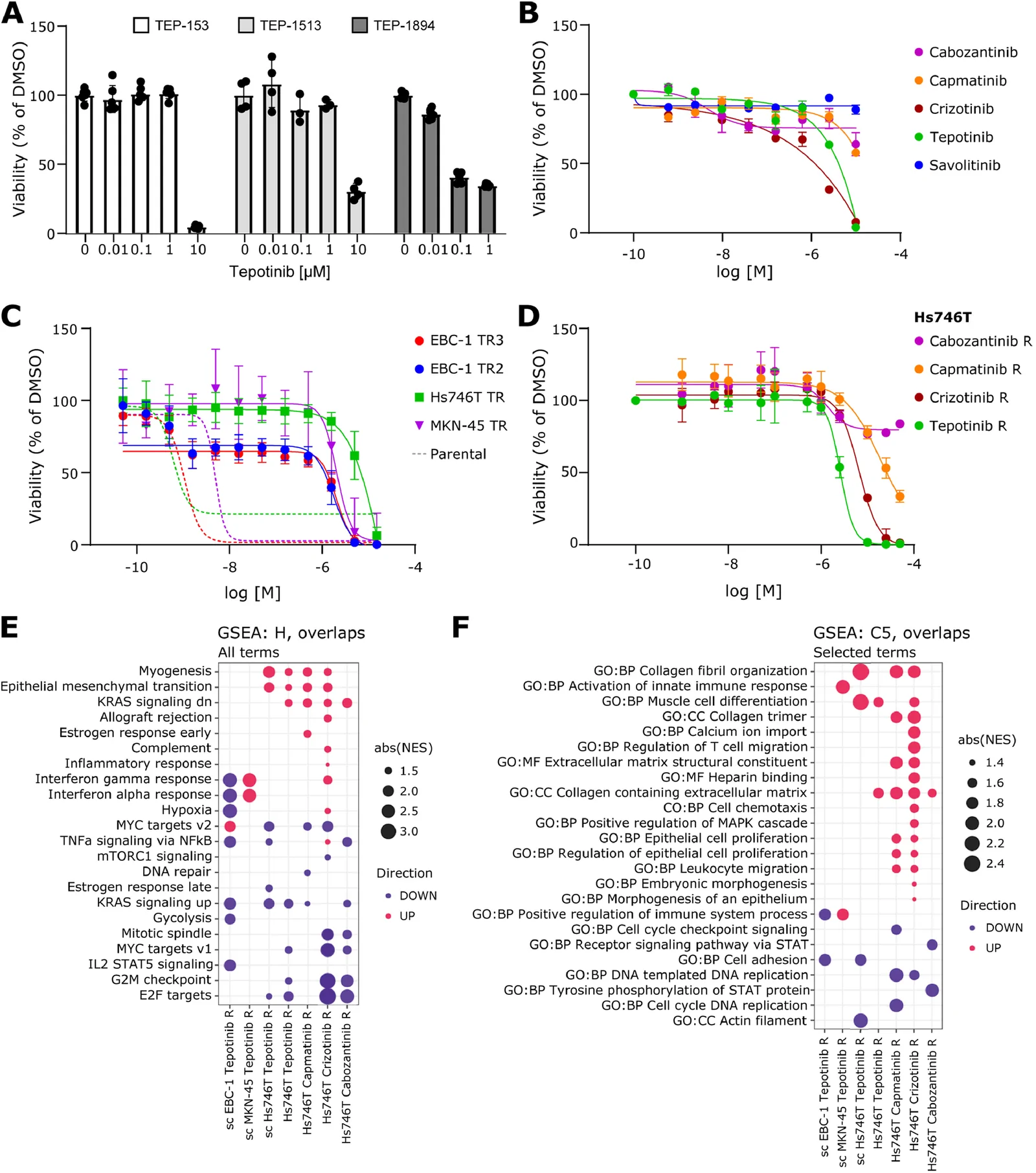
**Primary cell line-derived resistance models to MET inhibitors. A**, Resistance to tepotinib was confirmed by an MTT assay in three pleural effusion-derived primary cultures at disease progression to tepotinib after 72 hours of treatment. The 10 µM concentration was not assessed in TEP-1894, as sensitivity to tepotinib was observed around 100 nM. **B**, In TEP-153 primary cells, resistance to other METi was also assessed after 96 hours. **C**, Comparison of sensitivity to tepotinib in generated tepotinib-resistant cells (solid line), EBC-1, Hs746T, MKN-45, and their parental long-term culture control counterpart (dotted line). Two tepotinib-resistant EBC-1 clones were generated and evaluated. Viability was measured by CellTiter Glo after 96 hours of treatment. **D**, Hs746T cells resistant to cabozantinib, capmatinib, crizotinib, and another clone resistant to tepotinib were also generated, and resistance to each inhibitor was assessed and confirmed. Viability was measured by an MTT assay after 72 hours of treatment. **E-F**, Single-cell (sc) RNA sequencing of tepotinib-resistant cells and bulk RNA sequencing of METi-resistant Hs746T cells were performed. Resulting GSEA of hallmarks (H; **E**) and C5 (gene ontology; **F**) of 2-fold change DEGs was used to obtain normalized enrichment scores (NES), then compared across the different samples. The direction represents whether these terms go up or down in the resistant cells, compared to their parental counterpart. For C5, only selected terms are shown because the full output contained too many terms to display. An exploratory (non-confirmatory) p-value cutoff of p < 0.5 was used for H and C5 pathway enrichment, except for single-cell EBC-1 tepotinib-resistant data, where p < 0.25 was used, given the smaller sample size for this comparison.

Primary cultures established from tepotinib-progressing patients retained resistance to MET inhibition, exhibiting high IC_50_ values in the micromolar range (TEP-153 and TEP-1513; Fig. 3A). Resistance to other METi was tested in the TEP-153 primary culture, with no known genomic resistance mechanism, with similarly high IC_50_ values (Fig. 3B). Since no matched tepotinib-sensitive control was available for patient-derived cultures, METi-resistant cell lines were compared to their parental counterparts, resulting in 400- to 4000-fold decrease in tepotinib sensitivity (increased IC_50_ values; Fig. 3C). Hs746T cells resistant to cabozantinib (Type II), capmatinib (Type Ib), and crizotinib (Type Ia) similarly demonstrated IC_50_ values exceeding 1 µM (Fig. 3D).

Transcriptomic analyses demonstrated that *in vitro* resistant cell lines reproduced several biological programs identified in patient samples. Exploratory GSEA indicated enrichment of ECM-associated pathways, cell adhesion programs, and EMT-associated transcriptional signatures across multiple resistant models (Fig. 3E-F), consistent with the spatial transcriptomic analyses of progression biopsies.

The degree of concordance varied across METi-resistant cell lines. ECM-associated programs were noted across several models at exploratory thresholds, whereas immune-related pathways and other microenvironment-dependent signatures identified in patient tissues were only partially represented *in vitro*, reflecting the absence of a native tumor microenvironment. Several resistant cell-line models exhibited reduced KRAS signaling relative to their parental counterparts, whereas spatial transcriptomic analyses identified KRAS/MAPK pathway enrichment in two progressing tumors.

### Protein level analyses of MET inhibitor-resistant cells support partial EMT hypothesis

To determine whether EMT-associated transcriptional changes identified in METi-progressing patient samples and METi-resistant *in vitro* models were accompanied by corresponding protein-level alterations, we performed targeted protein analyses in resistant models.

Western blot analysis of EMT-related proteins demonstrated modest and heterogeneous changes across METi-resistant *in vitro* models (Supplementary Figure S3). E-cadherin increased in one EBC-1 tepotinib-resistant clone, while capmatinib- and crizotinib-resistant Hs746T cells exhibited slight increases in β-catenin. In contrast, vimentin levels did not increase upon resistance, suggesting that these resistant cell line models do not undergo canonical EMT despite the partial EMT-associated transcriptional adaptation detected by RNA-sequencing or in patient samples.

Protein expression of receptor tyrosine kinase pathway components (MET, EGFR, PI3K p110α, AKT, PTEN, MAPK, ERK) was similarly variable across METi-resistant cell lines (Supplementary Figure S3). MET expression decreased in tepotinib-resistant EBC-1 and Hs746T cells, whereas MET phosphorylation increased in cabozantinib-resistant Hs746T cells. Total AKT levels were upregulated in Hs746T cells upon resistance to all tested METi. Overall, expression and phosphorylation of signaling pathway components remained predominantly unchanged.

Given the heterogeneous genomic landscape and only partial EMT-associated adaptation observed in resistant models, we next investigated whether convergent functional dependencies emerge during acquired METi resistance.

### Genomic landscape of METi-resistance remains heterogeneous

Beyond transcriptional changes, genomic alterations may contribute to acquired METi resistance. To investigate this possibility, whole-exome sequencing (WES) was performed on patient-derived tepotinib-resistant primary cultures and *in vitro* generated METi-resistant cell lines using longitudinal tepotinib-sensitive baseline biopsies or long-term cultured parental cells as comparators. A summary of WES findings with potential biological relevance to METi resistance is provided in Supplementary Tables S3-5.

In patient-derived resistant cultures, bypass-associated amplifications of *KRAS* (TEP-1894) and *NRAS* (TEP-153) emerged upon tepotinib resistance. Additional amplifications included *SOX5, MDM2, PINX1,* and *SOX7*, while *CDKN2A* loss was detected in two patients, one accompanied by *MTAP* loss. SNVs and indels were similarly heterogeneous and included *BCLAF1*, C*DKN2A, CEACAM5, CEACAM8, EPHA5, FOXD1, RAD51B, RBM10,* and *USP14.* Eight genes (*GGT1, IGSF3, MADCAM1, OR4C5, POTED, PRDM9, PRSS3,* and *ZNF43*) were altered across the three resistant cultures. In METi-resistant cell lines, *KRAS* and *MDM2* amplifications and *CDKN2A* deletion were also observed, together with amplifications of *ALK, PIK3CA, RAF1, BRAF, EGFR, ERBB2, MAPK1*, or *TP53*.

A total of 544 unique resistance-associated genomic alterations were identified in resistant primary cultures or cell lines (98 genes in >1 sample, 16 genes in >2 samples). Functional annotation linked these alterations to calcium-dependent processes, DNA transcription, differentiation, actin-related functions, and ECM biology.

In summary, WES analyses identified diverse patient-specific and model-specific genomic alterations but failed to reveal a recurrent genomic resistance driver, reinforcing the heterogeneous genomic landscape of acquired METi resistance.

### Functional screening identifies shared METi-resistance-associated dependencies

**Fig. 4:**
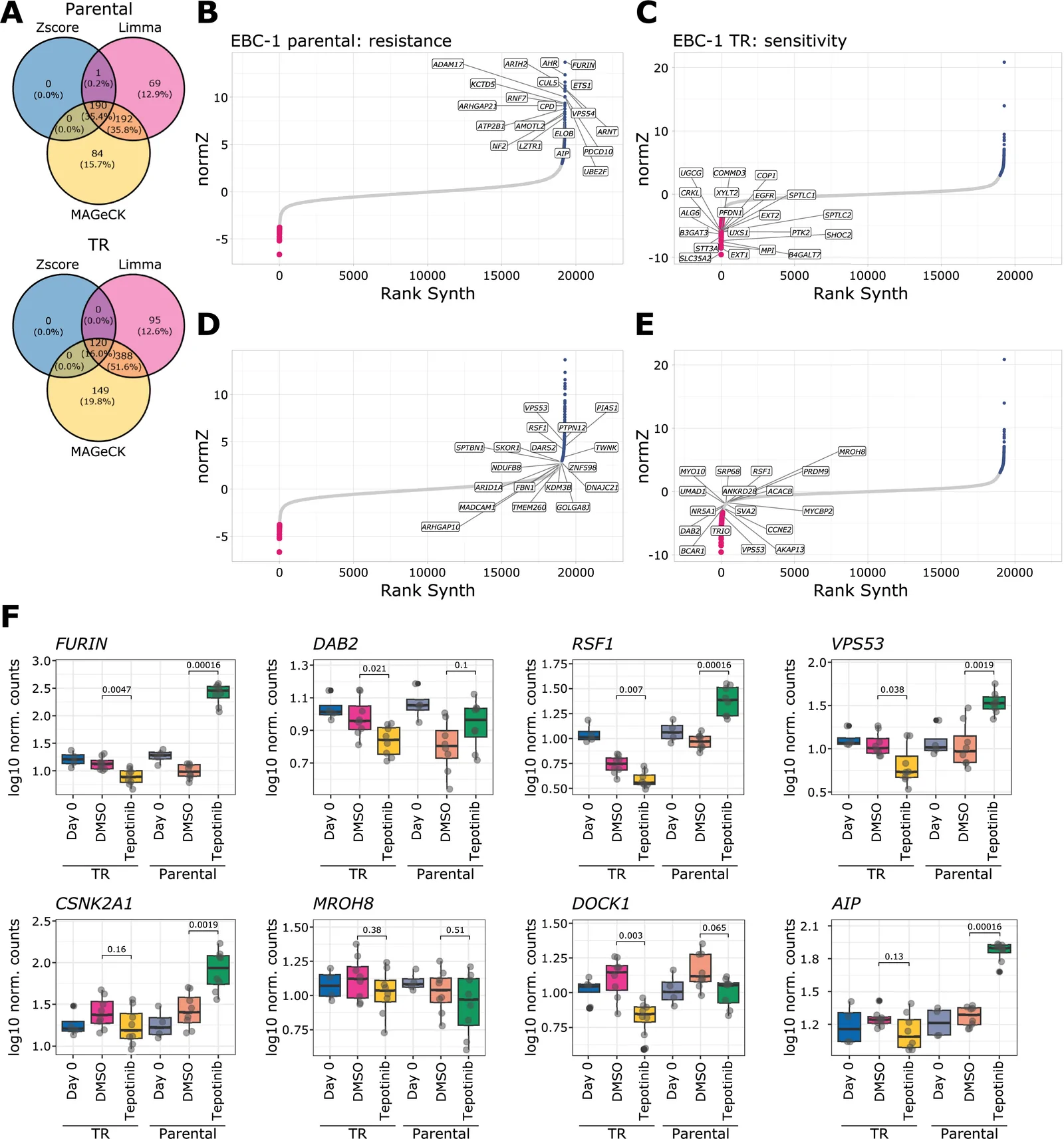
**Resistance and sensitivity CRISPR screens in parental and tepotinib-resistant EBC-1 cells reveal genes with potential roles in tepotinib resistance. A**, CRISPR screen results from EBC-1 parental resistance screen (top) and tepotinib-resistant (TR) EBC-1 sensitivity screen (bottom) using three analysis methods, Z-score, Limma, and MAGeCK. Genes overlapping in at least two methods were used for downstream analysis and comparisons. Top 20 genes from whole genome parental EBC-1 resistance CRISPR screen (**B**) and tepotinib-resistant EBC-1 sensitivity CRISPR screen (**C**) are annotated on Z-score plot of results. **D, E**, Overlaps between CRISPR screen results and WES SNVs from patients and all resistant cell lines observed are annotated on the CRISPR screen appropriate Z-score plot. **F**, Normalized counts of guide RNAs are shown for a subset of genes, with decreased counts in TR cells and increased counts in parental cells expected upon tepotinib treatment, should it play a role in sensitivity or resistance. P-values for DMSO versus tepotinib treatment are shown.

To identify functional dependencies associated with resistance, we performed complementary genome-wide CRISPR resistance and sensitivity screens in tepotinib-sensitive and tepotinib-resistant EBC-1 cells. Candidate genes were prioritized when identified by at least two independent analytical approaches (Fig. 4A). The top resistance-promoting and re-sensitizing genes are shown in Fig. 4B-C, while CRISPR screen quality metrics are provided in Supplementary Figure S4.

EGFR emerged as a top candidate and was additionally amplified in resistant cell lines. Concordance between CRISPR screening and WES findings was observed for several candidates and is summarized in Fig. 4D–E. Representative guide RNA enrichment and depletion profiles are shown for CRISPR-specific hits (*FURIN, CSNK2A1, DOCK1*, and *AIP*), genes identified by both CRISPR screening and WES (*RSF1* and *VPS53*), and genes supported by CRISPR, WES, and RNA-seq analyses (*DAB2* and *MROH8*; Fig. 4F).

Together, these findings indicate that although resistance-associated genomic alterations are highly heterogeneous, common functional candidates emerge across independent screening approaches, suggesting that diverse genomic resistance trajectories may share a more limited, overlapping set of biological dependencies.

### Overlapping therapeutic vulnerabilities emerge across METi-resistant models

**Fig. 5:**
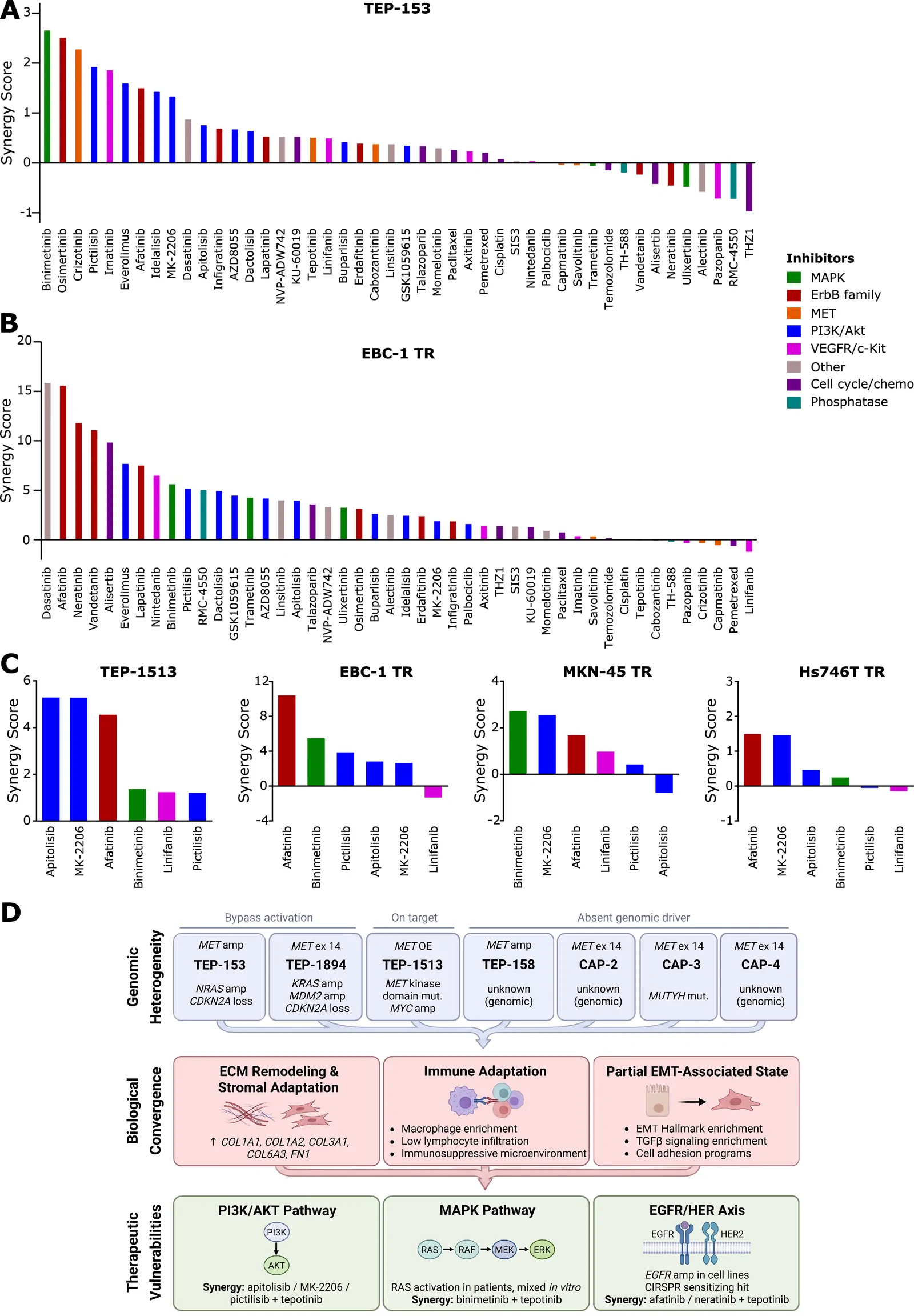
**Combination drug screening and integrated resistance modeling reveal shared therapeutic vulnerabilities.** Synergy scores of tepotinib combined with inhibitors of MAPK, ErbB family, MET, PI3K/Akt, VEGFR/c-Kit, cell cycle, phosphatase, or other inhibitors (x-axis) were determined after 96 hours of combination treatment. **A**, A large screen was first performed in the patient progression-derived TEP-153 cells, and **B**, *in vitro*-generated EBC-1 tepotinib-resistant (TR) clones (combined results from 2 clones). **C**, Six of the top hits were then selected for testing in remaining tepotinib-resistant cells. **D***,* Summary schema of patient genomic heterogeneity at METi resistance, linked by shared biological programs and resulting in therapeutic vulnerabilities (created with Biorender.com).

To determine whether biologically shared resistance programs translate into shared therapeutic dependencies, we performed combination drug screening with tepotinib in resistant primary cultures and METi-resistant cell lines. Initial assessment in the TEP-153 primary culture (lacking *MET* kinase domain mutations, with *NRAS* amplification) demonstrated synergistic interactions between tepotinib and inhibitors targeting MAPK, EGFR/HER2, or PI3K/Akt signaling pathways (Fig. 5A). Among these combinations, the MEK inhibitor binimetinib demonstrated the strongest synergistic activity.

Given the transcriptional similarities observed between resistant primary cultures and resistant cell-line models, combination screening was extended to two tepotinib-resistant EBC-1 clones. These studies confirmed synergistic interactions between tepotinib and inhibitors targeting MAPK, EGFR/HER2, and PI3K/Akt signaling pathways, although the relative strength of individual combinations differed across models (Figure 5B; averaged). Monotherapy sensitivity (IC_50_ value) remained largely unchanged, except for resistance to METi and increased sensitivity to dasatinib and neratinib (Supplementary Figure S5).

Six leading combinations were subsequently evaluated in TEP-1513 primary cells harboring multiple *MET* kinase domain mutations (p.D1228N/H and p.Y1230H) and in additional tepotinib-resistant models (Fig. 5C). In TEP-1513 cells, apitolisib, MK-2206, and afatinib showed synergistic activity when combined with tepotinib. In EBC-1 tepotinib-resistant cells, larger screen results were confirmed, while synergy was less pronounced in other tepotinib-resistant cell lines. Nevertheless, recurrent synergistic interactions involving EGFR/HER2 and PI3K pathway inhibition were observed across distinct resistance contexts, supporting the existence of shared therapeutic dependencies despite heterogeneous resistance-associated genomic alterations.

Collectively, these findings indicate that acquired METi resistance converges on functionally targetable signaling programs. Although resistant tumors and models exhibit diverse genomic resistance mechanisms, recurrent vulnerabilities involving MAPK, EGFR/HER2, and PI3K signaling emerge across multiple resistance contexts. These shared vulnerabilities point to overlapping therapeutic dependencies despite genomic heterogeneity (summary schema in Fig. 5D).

## Discussion

In this study, we investigated resistance to MET inhibitors in MET-altered NSCLC using paired patient samples, patient-derived cultures, and resistant cell-line models. The cohort included *MET*ex14 skipping, *MET*amp, and MET-OE, which differ in MET dependency and clinical response to METi. Given the scarcity of paired post-progression samples, we analyzed these biologically distinct subsets together, while interpreting conclusions cautiously. Across cases, three main routes of resistance emerged: [1] on-target MET evolution, [2] ECM/TME remodeling with partial EMT–related transcriptional programs and immune-context shifts, and [3] bypass signaling via EGFR/HER, MAPK, and PI3K/Akt. These findings support a hypothesis-generating framework rather than universal mechanisms for all MET-altered NSCLC.

*MET* kinase domain mutations remain a direct on-target resistance mechanism [18], while RAS-MAPK and EGFR/HER activation can bypass MET inhibition through alternative signaling pathways [25,[33], [34], [35]]. Recent clinical reports further support this model, showing that secondary MET D1228 and Y1230 mutations can emerge after type I MET inhibitors and may remain sensitive to type II MET inhibitors such as cabozantinib or savolitinib in selected cases [36,37]. However, on-target alterations did not explain resistance across all samples. This highlights the need to move beyond single-mutation resistance models and to incorporate pathway-level and microenvironmental readouts into future biomarker strategies.

Spatial transcriptomics identified ECM remodeling as a shared feature of resistant samples, including upregulation of collagen genes and *FN1*. Together with the *in vitro* data, these findings suggest that ECM remodeling may contribute to METi resistance by influencing adhesion, survival signaling, matrix structure, immune exclusion, and TME interactions, consistent with prior links between ECM remodeling, tumor progression, immune escape, and therapy resistance [38]. The resistance of TEP-153 in the absence of a detectable *MET* kinase domain mutation further supports the possibility that non-on-target mechanisms can contribute to METi resistance.

Resistant samples also showed low lymphocyte infiltration with macrophage-associated and complement-related signatures, consistent with an immunosuppressive microenvironment. This contrasts with our prior observation of a more immuno-permissive TME during response to neoadjuvant tepotinib [30], suggesting that immune context may differ between response and acquired resistance. Together with previous reports linking MET-altered NSCLC to immune-suppressed or immune-activated subtypes [33], these findings support a context-dependent role of the TME in METi resistance.

EMT-associated transcriptional signatures should be interpreted cautiously. Although RNA sequencing and spatial transcriptomics suggested EMT-related programs, Western blot analyses showed only modest changes in E-cadherin, β-catenin, and vimentin. Thus, these signatures likely reflect partial EMT, ECM/stromal contributions, or microenvironment-driven states rather than complete canonical EMT in all resistant tumor cells. The simplicity of *in vitro* systems may also explain why transcriptional EMT-related changes were not accompanied by strong protein-level evidence of EMT. Partial EMT in this study is therefore best viewed as one component of a broader resistant-state program involving ECM remodeling, cytoskeletal dynamics, cell adhesion, and TME interactions.

KRAS/MAPK signaling also appeared context dependent. Patient samples showed KRAS/MAPK activation in selected cases, whereas several resistant *in vitro* models showed reduced KRAS signaling. This discrepancy should not be interpreted as proof that the TME drives KRAS/MAPK activation, but rather as evidence that homogeneous *in vitro* systems incompletely capture clinical resistance. Future co-culture, conditioned-media, *ex vivo*, or TME-containing models will be needed to test this directly.

The *in vitro* models provided a useful platform to explore tumor-cell-intrinsic resistance programs and pharmacologic vulnerabilities, but they also have important limitations. Hs746T and MKN-45 are gastric cancer cell lines, and tissue-of-origin differences may influence MET dependency, ECM signaling, and resistance biology compared with NSCLC. Moreover, cell-line models lack immune architecture, stromal organization, spatial structure, and dynamic TME interactions. Pathway-enrichment findings in these models relied on a lenient, exploratory p-value threshold rather than a stringent adjusted cutoff and should be viewed as hypothesis-generating rather than confirmatory. Thus, while these models reproduced several ECM-associated, adhesion-related, EMT-associated, and bypass-signaling features of resistance, they should be interpreted as mechanistic tools rather than complete models of clinical METi resistance.

Although genomic alterations were highly diverse, genome-wide CRISPR screens identified overlapping functional dependencies, suggesting that shared biology may exist at the level of pathway requirement rather than mutational recurrence. The functional data support rational combination strategies tailored to molecular context. In TEP-153, which lacked detectable *MET* kinase domain mutations but showed *NRAS* amplification, tepotinib combined with MAPK inhibition showed strong synergy. In TEP-1513, which harbored *MET* kinase domain mutations, PI3K/Akt- and EGFR-directed combinations showed activity. These findings suggest that combined MET and EGFR/HER, MAPK/MEK, or PI3K/Akt inhibition may overcome selected resistance states, although these strategies require validation in larger and more clinically representative models. Partial EMT and ECM remodeling may contribute to the resistant phenotype, but they may also reflect downstream consequences of upstream signaling rewiring; therefore, proximal bypass pathways may represent more immediately actionable therapeutic targets.

Overall, this study integrates spatial transcriptomics, WES, RNA sequencing, CRISPR screening, protein analyses, and pharmacologic testing to connect genomic evolution with spatially resolved transcriptomic and functional resistance programs. The major conceptual model emerging from these data is that METi resistance in MET-altered NSCLC is not explained by a single recurrent event, but by overlapping and context-dependent programs involving on-target MET evolution, bypass signaling, ECM/TME remodeling, partial EMT-associated transcriptional states, and altered immune context. These findings provide a hypothesis-generating framework for future biomarker development and rational combination strategies in MET-altered NSCLC.

## Conclusion

Altogether, our results show that resistance to MET inhibitors in MET-altered NSCLC is heterogeneous and cannot be explained by a single mechanism. Instead, multiple interconnected factors such as on-target *MET* kinase domain mutations, changes in the extracellular matrix and tumor microenvironment, partial EMT-related changes, and activation of bypass pathways such as EGFR/HER, RAS-MAPK, and PI3K/Akt consistently emerged in the METi-resistant setting. Future studies integrating tissue, liquid biopsy, and multi-omics data with robust *ex vivo* and *in vivo* models will be essential to better define METi resistance and guide rational therapeutic strategies.

## CRediT authorship contribution statement

**Manon A. Simard:** Writing – review & editing, Writing – original draft, Visualization, Validation, Supervision, Software, Resources, Project administration, Methodology, Investigation, Formal analysis, Data curation, Conceptualization. **Elba Marín:** Writing – review & editing, Writing – original draft, Visualization, Validation, Supervision, Software, Resources, Methodology, Investigation, Funding acquisition, Formal analysis, Data curation, Conceptualization. **Silvia García-Roman:** Writing – review & editing, Formal analysis, Data curation. **Cristina Aguado:** Writing – review & editing, Formal analysis, Data curation. **Michail Yekelchyk:** Writing – review & editing, Visualization, Software, Formal analysis, Data curation. **Carolin Schmelas:** Writing – review & editing, Visualization, Methodology, Investigation, Funding acquisition, Formal analysis, Data curation, Conceptualization. **Nina Berges:** Writing – review & editing, Resources, Investigation, Formal analysis, Data curation, Conceptualization. **Balca R. Mardin:** Writing – review & editing, Visualization, Software, Resources, Investigation, Formal analysis, Data curation. **Sophie Ockfen:** Writing – review & editing, Data curation. **Rebecca Schaefer:** Writing – review & editing, Data curation. **Markus Esser:** Writing – review & editing, Data curation. **Jing Yang:** Writing – review & editing, Visualization, Software, Formal analysis, Data curation. **Felix Geist:** Writing – review & editing, Software, Formal analysis. **Oliver Schadt:** Writing – review & editing. **Christof Reusch:** Writing – review & editing, Investigation, Data curation. **Sebastian Bender:** Writing – review & editing, Data curation. **Núria Jordana-Ariza:** Writing – review & editing, Investigation. **Carlos Esparré:** Writing – review & editing, Investigation. **Ruth Román:** Writing – review & editing, Investigation. **Tolulope Apata:** Writing – review & editing, Investigation. **Cristina Teixido:** Writing – review & editing, Validation, Supervision, Investigation. **Marc Rico-Pastó:** Writing – review & editing, Software, Investigation. **Sara Hijazo-Pechero:** Writing – review & editing, Software, Investigation. **Marina Querol:** Writing – review & editing. **Marie Morfouace:** Writing – review & editing, Supervision, Project administration. **Miguel Ángel Molina-Vila:** Writing – review & editing, Writing – original draft, Visualization, Validation, Supervision, Resources, Formal analysis, Data curation, Conceptualization. **Noemí Reguart:** Writing – review & editing, Writing – original draft, Visualization, Validation, Supervision, Resources, Funding acquisition, Formal analysis, Data curation, Conceptualization. **Niki Karachaliou:** Writing – review & editing, Writing – original draft, Visualization, Validation, Supervision, Resources, Project administration, Formal analysis, Data curation, Conceptualization.

## Declaration of competing interest

Pangaea Oncology received funding from the healthcare business of Merck KGaA, Darmstadt, Germany for part of this work. MA Simard, M Yekelchyk, C Schmelas, N Berges, BR Mardin, S Ockfen, R Schaefer, M Esser, J Yang, F Geist, C Reusch, S Bender, and N Karachaliou are employees of the healthcare business of Merck KGaA, Darmstadt, Germany. M Morfouace was an employee of the healthcare business of Merck KGaA, Darmstadt, Germany at the time of her involvement. S Ockfen, R Schaefer, and O Schadt own stock in the healthcare business of Merck KGaA, Darmstadt, Germany. C Teixido has consulted for AstraZeneca, Merck & Co., Inc., Rahway, NJ, Novartis, Biocartis; lectures and educational activities for AstraZeneca, Roche, Janssen, Pfizer, Biocartis; received research grants from Novartis and AstraZeneca; and received travel, accommodations, expenses from Lilly and Merck & Co., Inc., Rahway, NJ. N Reguart has served on advisor boards for AbbVie, Amgen, Astra Zeneca, Bayer, BMS, Boehringer, Gilead, Harpoon, Janssen, Merck & Co., Inc., Rahway, NJ, Novartis, Pfizer, Regeneron, Revolution Medicine, Roche, Sanofi, Summit Therapeutics, Takeda, and Tubulis, has provided expert testimony for Janssen and the healthcare business of Merck KGaA, Darmstadt, Germany, and has been an invited speaker at Amgen, Astra Zeneca, BMS, Johnson & Johnson, the healthcare business of Merck KGaA, Darmstadt, Germany, Merck & Co., Inc., Rahway, NJ, Novartis, PharmaMar, and Sanofi. In addition, N Reguart is the Principal Investigator of the PEERS Investigator Initiated Trial sponsored by Merck & Co., Inc., Rahway, NJ, is a member of the ArriVent Biopharma steering committee, and is involved in writing a manuscript with Amgen.
