# Supplementary material for: Understanding tumor adaptations and resistance to MET inhibitors in MET-altered non-small cell lung cancer
Source: Neoplasia. 2026 Sep 14;81:101359. doi: 10.1016/j.neo.2026.101359 (PMC13594760; doi:10.1016/j.neo.2026.101359)
Supplement: Supplementary file 1 [file mmc1.docx]

# **Supplementary Material**

## **Supplementary Methods**

**SM.1	Spatial Transcriptomics**

The Solid Tumor TME panel from Bruker Spatial Biology (NanoString) was used, including the SYTO 13 nuclear stain, PanCK and CD45. The Cancer Transcriptome Atlas (CTA) RNA probes were used for spatial transcriptomics supplemented with custom probes for *MET*ex14 skipping generated by Bruker Spatial Biology (NanoString). Circular ROIs of 300 μm diameter were selected with pathologist guidance to capture high-quality regions representing the tissue heterogeneity (3-23 ROI per sample, depending on sample size) and were segmented in tumor and microenvironment compartments, based on CD45 staining.

Samples were sequenced using an Illumina NovaSeq 6000 at Medicover IMGM Laboratories (Mansfried, Germany). Sequencing data was processed in the GeoMx analysis suite (v3.1.0; NanoString, Bruker) and normalized using a third quartile (Q3) normalization. Comparisons between samples and groups were performed using linear mixed models with Benjamini-Hochberg correction. Scan date, sample, and ROI were included as random effects, accounting for the nested structure of multiple ROIs within each sample, with each sample corresponding to one patient at one timepoint (pre-treatment or progression). Gene set enrichment analysis for hallmarks and C5 (Gene Ontology) collections from the Molecular Signatures Database (MSigDB) were performed downstream in R. Selection of C5 terms was performed based on biological acquired resistance relevance and recurrent programs across samples. The Wilcoxon Test was used to calculate p-values for pairwise comparisons (1). An adjusted p-value <0.05 was used as the significance threshold.

**SM.2	Next-Generation Sequencing**

DNA-based NGS was performed on samples from patients treated with tepotinib and the derived primary cultures using the GeneRead® QIAact Custom extended Panel (Qiagen, Hilden, Germany). which detects mutations and copy number variations in *EGFR, BRAF, MET, ERBB2, ALK, ROS1, PIK3CA, KRAS, NRAS, FGFR1, FGFR2, FGFR3, KIT, PDGFRA, TP53, CDK4, CDK6, IDH1, IDH2, ERBB4, STK11, MYC, RET, POLE, POLD1, KEAP1, ARID1A, FAT1, NFE2L2,* and *SETD2*. Clonal amplification was performed on pooled libraries and, following bead enrichment, the GeneReader instrument was used for sequencing. Qiagen Clinical Insight Analyze software was employed to align the read data and call sequence variants, with the Qiagen Clinical Insight Interpret web interface for data interpretation.

NGS was performed on samples from patients treated with capmatinib using the commercially available Oncomine Solid Tumor panel (Thermo Fisher Scientific), Oncomine Focus panel (Thermo Fisher Scientific), and/or Foundation One (Foundation Medicine) for tissues and Guardant (Guardant Health), Foundation One (Foundation Medicine), or InVision First (NeoGenomics Laboratories) for blood. This, in part, was used to identify *MET* amplification or *MET* exon 14 skipping alterations.

**SM.3	RNA Sequencing Analysis**

For RNA-seq analysis, quality control checks were performed using FastQC version 0.11.4 (2). Adapter removal and trimming of low-quality quality reads (Q<30) was performed using fastp version 0.21 (3). Alignment of processed reads was performed using STAR version 2.6.0 (3) using GENCODE release 33 (4) as the reference genome. Quantification of aligned reads to raw counts was done with RSEM version 1.3.1 (5). R 4.1.1 and RStudio were used for downstream analysis using tidyverse (2.0.0), qs (0.26.1), plyr (1.8.9), ggpubr (0.6.0), patchwork (1.2.0), Seurat (5.0.3), edgeR (4.4.2), Limma (3.62.2), PCAtools (2.18.0), factoextra (1.0.7), msigdb (7.5.1), clusterProfiler (4.14.4), fgsea (1.32.2), the latter two for GSEA using the C5 (Gene Ontology) and the H (Hallmark) collections from MSigDB (6-11). Selection of C5 terms was performed based on biological acquired resistance relevance and recurrent programs across samples. The Wilcoxon Test was used to calculate p-values for pairwise comparisons (1). A less stringent adjusted p-value cutoff (p<0.5, or p<0.25 for single-cell EBC-1 tepotinib-resistant data) was applied given the smaller sample sizes and higher technical noise of these comparisons; results at these thresholds are considered exploratory and hypothesis-generating rather than confirmatory, and are distinguished accordingly in the text.(12).

**SM.4	Whole Exome Sequencing**

For patient samples, variants were identified computationally using a variety of bioinformatics tools (GATK Unified Genotyper, samtools mpileup, SHORE, Annovar, Indelocator). Three independent predictions were combined and filtered with two additional tools (“GATK Variant Filtration” “GATK combine Variants”). Databases such as Clinvar, HGMD Professional, OMIM, Orphanet, and NCBI databases and predictor bioinformatics tools, such as VarSome or own databases and tools, were used to filter the final variants, eliminating the SNVs with rs identifier and population frequencies >0.005. Only SNVs and small indels with allelic fractions >0.25 were considered.

For METi-resistant cell lines and their parental counterparts, FASTQ files were uploaded to the Mercury^TM^ Software (IntegraGen, France) for the identification of SNVs and indels, as well as copy number variations (CNV). For the interpretation of WES results, PubMed was used for scientific literature searches, Clinvar for annotation of variants (13), PolyPhen-2 (14, 15) and Combined Annotation Dependent Depletion (16, 17) for prediction of biological impact, COSMICv99 (18, 19) for finding mutation frequencies and KEGG pathways in cancer (20), and STRING (21) for pathway analysis.

**SM.5	CRISPR screen**

*SM.5.1	Cas9 Cell Line Generation*

The genome-wide lentiviral CRISPR KO library generated by Cellecta (KOHGW-80Kv2-P). Lentiviral particles were produced by co-transfecting 293FT cells (Life Technologies GmbH, R70007) with the genome-wide library construct in the pRSG16-U6-sg-UbiC-RFP-puro construct (Cellecta), pMD2.G and psPAX2 plasmids using Lipofectamine 3000 (Thermo Fisher Scientific, L3000001). At 48 hours post-transfection, viral supernatant was collected and filtered through a vacuum filtration system with 0.22 µm filter (PES-polyether sulfone, low protein-binding) from Millipore (S2GPU05RE), then concentrated 100x with Lenti-X Concentrator (Takara Bio, 631232).

Tepotinib-sensitive parental EBC-1 cells and one variant of tepotinib-resistant EBC-1 cells (R5B-II.2, TR2) were transduced with (Edit-R Inducible Lentiviral hEF1aBlast-Cas9 from Horizon Discovery Biosciences, VCAS11227). Single cell clones were generated by serial dilution, and Cas9 induction was verified by Western Blot and functional assay that comprised the transfection of a gRNA against the essential gene POLR2A (Supplementary Fig. S5A, B). Sensitivity and resistance against tepotinib were re-evaluated for the parental EBC-1 (clone 10) as well as EBC-1 TR2 (clone 5), respectively.

*SM.5.2	Tepotinib Resistance and Sensitivity Screens*

For the tepotinib-resistance screen, 55 million tepotinib-sensitive EBC-1 iCas9 cells (clone 10) were aliquoted to 800,000 cells per well of a 6-well plate, while for the tepotinib-sensitivity screen, 35 million tepotinib-resistant EBC-1 iCas9 cells (clone 5) were aliquoted in 500,000 cells per well of a 6-well plate. The cells in the 6-well plates were spinfected with the lentiviral library at 2,000 rpm, for 2 h at 37°C, with the addition of 8 μg/mL polybrene (Sigma-Aldrich, TR-1003) to reach a multiplicity of infection of 0.3. Cells were left to recover in fresh medium for 24 h incubated at 37°C, 5% CO2. The infected cells were selected with 1 μg/mL puromycin (Thermo Fisher Scientific, A1113803) for 3 days, following which, Cas9 was induced (1 μg/mL doxycycline; Day 0) for 7 days before treatment with either DMSO or tepotinib. For the resistance screen, cells were treated with 3.63 nM tepotinib for 3 days (Day 7 to Day 10) and reached IC_70_. The media was replaced with fresh medium after a single treatment, and cells were harvested on Day 17. For the sensitivity screen, EBC-1 TR2 clone 5 was treated with 50 nM tepotinib every 3 to 4 days for a total of three consecutive treatment rounds and reached IC_50_. Cells were harvested immediately after the last treatment on Day 18.

For preparation of the sequencing libraries, genomic DNA was isolated from the initial and final time points using QIAamp DNA Blood Kit (Qiagen, 51106). Twenty-four separate polymerase chain reaction (PCR) reactions using plasmid-specific primers for the amplification of the gRNA coding sequences were performed, then pooled and used as a template for a second PCR amplification, divided into eight PCRs for the addition of Illumina adaptors and sample-specific barcodes. Q5 NEBNext Hot Start polymerase master mix (M0543) was used for PCR reactions. PCR reaction products of 390 bp were purified using 0.8X Agencourt AMPure XP (Beckman Coulter, A63880) and eluted in low EDTA TE buffer (Thermo Fisher, 12,090,015). All samples were pooled and sequenced using Illumina NextSeq2000 platform (Illumina), using 75-base single reads.

Cell fitness was calculated based on the gRNA counts at the beginning and end of the experiment. Three independent methods were used to count gRNA numbers from each screen and for downstream analyses: DrugZ (22), MAGeCK (23), and Limma (6). We classified hits as genes identified to be significantly depleted in two out of the three analysis methods.

**SM.6	Western blotting**

A total of 25–70 μg of protein were used for assessing the expression of MET (25H2; Cell Signaling, 3127), phospho-Y1234/1235 MET (D26; Cell Signaling, 3077), p38 MAPK (Cell Signaling, 9212), phospho-T180/Y182 p38 MAPK (Cell Signaling, 9211), AKT (40D4; Cell Signaling, 2920), phospho-S473 AKT (D9E, Cell Signaling 4060), EGFR (1F4; Cell Signaling, 2239), phospho-Y1068 EGFR (Invitrogen, 44788), PTEN (138G6; Cell Signaling, 9559), PI3K p100α (C73F8; Cell Signaling, 4249), E-cadherin (5H6L18; Invitrogen, 701134), β-catenin (D10A8; Cell Signaling, 8480), and vimentin (J144; Pierce, MA3-745) by Western blot. Secondary antibodies IRDye anti-Rabbit 800 CW (Licor Biosciences, 925-32211) or IRDye anti-Mouse 800 CW (Licor Biosciences, 926-32210) were used. The immunoreactive bands were quantified using ImageJ software (v1.53), and the resulting data were analyzed in GraphPad Prism 10.0 to assess significant differences in protein expression levels among the different cellular models.

## **Supplementary Tables**

Supplementary Table S1**: Inhibitors for synergy testing.**

| **Inhibitor** | **Source** | **Concentration range** | **Inhibitor** | **Source** | **Concentration range** |
| --- | --- | --- | --- | --- | --- |
| Afatinib | Chem Shuttle | 5 nM – 15 μM | Linsitinib | Angene Chemical | 5 nM – 15 μM |
| Alectinib | BOC Sciences | 5 nM – 15 μM | MK-2206 | Chemie Tek | 5 nM – 15 μM |
| Alisertib | Selleck Chemicals | 0.5 nM – 1.58 μM | Momelotinib | Selleck Chemicals | 5 nM – 15 μM |
| Apitolisib | Active Biochem | 0.5 nM – 1.58 μM | Neratinib | Activate Scientific | 5 nM – 15 μM |
| Axitinib | Selleck Chemicals | 5 nM – 15 μM | Nintedanib | Key Organics | 5 nM – 15 μM |
| AZD8055 | Selleck Chemicals | 0.5 nM – 1.58 μM | NVP-ADW-742 | In-house synthesis | 5 nM – 15 μM |
| Binimetinib | PharmBlock | 0.16 nM – 0.5 μM | Osimertinib | ABCR | 5 nM – 15 μM |
| Buparlisib | ChemLin Chemical | 5 nM – 15 μM | Paclitaxel | VWR | 4 pM – 16 nM |
| Cabozantinib | MedChem Express | 5 nM – 15 μM | Palbociclib | ABCR | 5 nM – 15 μM |
| Capmatinib | Chem Shuttle | 5 nM – 15 μM | Pazopanib | Selleck Chemicals | 5 nM – 15 μM |
| Cisplatin | Sigma-Aldrich | 5 nM – 15 μM | Pemetrexed | ABCR | 5 nM – 15 μM |
| Crizotinib | Chem Shuttle | 5 nM – 15 μM | Pictilisib | Selleck Chemicals | 5 nM – 15 μM |
| Dactolisib | Selleck Chemicals | 1.7 nM – 5 μM | RMC-4550 | Sigma-Aldrich | 5 nM – 15 μM |
| Dasatinib | BLD Pharmatech | 5 nM – 15 μM | Savolitinib | Enamine | 5 nM – 15 μM |
| Erdafinitib | Astatech | 5 nM – 15 μM | SIS3 | Santa Cruz Biotechnology | 5 nM – 15 μM |
| Everolimus | Selleck Chemicals | 46 pM – 158 nM | Talazoparib | Haoyuan ChemExpress | 5 nM – 15 μM |
| GSK1059615 | Custom synthesis | 5 nM – 15 μM | Temozolomide | Pharmeks | 5 nM – 15 μM |
| Idelalisib | Syngene | 5 nM – 15 μM | Tepotinib | In-house synthesis | 5 nM – 15 μM |
| Imatinib | ABCR | 5 nM – 15 μM | TH-588 | In-house synthesis | 5 nM – 15 μM |
| Infigratinib | Axon MedChem | 5 nM – 15 μM | THZ1 | Target Mol | 5 nM – 15 μM |
| KU-60019 | MedChem Express | 5 nM – 15 μM | Trametinib | Boaopharma | 5 pM – 15.8 nM |
| Lapatinib | AOK Chem | 5 nM – 15 μM | Ulixertinib | BLD Pharmatech | 0.5 nM – 1.58 μM |
| Linifanib | Selleck Chemicals | 5 nM – 15 μM | Vandetanib | MercaChem | 5 nM – 15 μM |

Supplementary Table S2: **MET alteration and co-alterations in patients treated with MET inhibitors.**

| Patient # | Method | Presentation | Progression | Primary culture |
| --- | --- | --- | --- | --- |
| TEP-153 | nCounter | N/A | MET OE | MET OE |
|  | NGS | *MET*: amp *TP53*: c.993+2T>G, 23%  *BRAF*: loss | *MET*: amp *TP53*: c.993+2T>G, 65% *ARID1A*: p.N2220fs*11, 77% | *MET*: amp *TP53*: c.993+2T>G, 49% *ARID1A*: p.N2220fs*11, 62% |
| TEP-158 | nCounter | MET OE | FFPE: Negative | N/A |
|  | NGS | *MET*: amp (14 copies) *MYC*: amp (15 copies) *STK11*: c.921-1G>T, 36% *TP53*: p.V272L, 42% | Blood *MET*: amp & p.D1228N, 16% *STK11*: C921-1G>T, 4% *TP53*: p.V272L, 5% FFPE *MET*: amp (12 copies) *STK11*: c921-1G>T, 10% *TP53*: p.V272L, 36% | N/A |
| TEP-1513 | nCounter | MET OE | MET OE | MET OE |
|  | NGS | *TP53*: p.R213*, 21% *FAT1*: p.G2653S, 50% *FGFR1*: c.2186+9G>A, 49% | *MET*: p.D1228N, 27%; p.D1228H, 1%; p.Y1230H, 6% *TP53*: p.R213*, 86% *FAT1*: p.G2653S, 46% *FGFR1*: c.2186+9G>A, 57% | *MET*: amp & p.D1228N, 28%; p.D1228H, 1%; p.Y1230H, 7% *TP53*: p.R213*, 81% *FAT1*: p.G2653S, 47% *FGFR1*: c.2186+9G>A, 55% *MYC*: amp |
| TEP-1894^a^ | nCounter | *MET*ex14 skipping | *MET*ex14 skipping | *MET*ex14 skipping, MET OE, KRAS OE |
|  | NGS | *MET*: exon 14,  p.D1010H, 35% *ERBB2*: p.A371D, 45% | *MET*: p.D1010H, 48% *KRAS*: amp | *MET*: amp (6 copies) & p.D1010H, 89% *KRAS*: amp (73 copies) |
| CAP-2 | nCounter | *MET*ex14 skipping | N/A | N/A |
|  | NGS | N/A | N/A | N/A |
| CAP-3 | nCounter | *MET*ex14 skipping | N/A | N/A |
|  | NGS | *TP53*: p.H193Y *MET*: amp | *MET*ex14 skipping *TP53*: p.H193Y *MUTYH*: p.G382D | N/A |
| CAP-4 | nCounter | N/A | N/A | N/A |
|  | NGS | *MET*ex14 skipping | *MET*ex14 skipping | N/A |

^a^*N.B. For TEP-1894, WES instead of NGS was performed and results from genes included in NGS are represented here.*

Supplementary Table S3: **Genetic alterations detected by WES in patients.**

| Patient | Genetic alteration | Only at presentation | Only in progression primary culture | In both |
| --- | --- | --- | --- | --- |
| TEP-153 | SNVs and indels | 104 | 175 | 758 |
|  | CNVs | 10 | 10 | 2 |
| TEP-1513 | SNVs and indels | 300 | 148 | 546 |
|  | CNVs | 65 | 330 | 6 |
| TEP-1894 | SNVs and indels | 77 | 256 | 489 |
|  | CNVs | 58 | 443 | 40 |

Supplementary Table S4**: Cancer-relevant CNVs detected only at resistance by WES.**

|  | **Chr** | **Gene** | **Type** | **Sample (copies)** |
| --- | --- | --- | --- | --- |
| Patient only | 1 | *NRAS* | Amplification | TEP-153 (37) |
|  | 8 | *PINX1, SOX7* | Amplification | TEP-1894 (18) |
|  | 8 | *MYC* | Amplification | TEP-1513 (3-4) |
|  | 20 | *STK4* | Deletion | TEP-1513 |
|  | X | *STAG2* | Deletion | TEP-1894 (0) |
| Patient and cell line | 7 | *MET^a^* | Amplification | TEP-153 (75), TEP-1894 (6.0), EBC-1 TR3 (54), MKN-45 TR (6.2), Hs746T TR (140) |
|  | 9 | *CDKN2A* | Deletion | TEP-153 (0), TEP-1894 (0), MKN-45 TR (0) |
|  | 12 | *KRAS* | Amplification | TEP-1894 (70), EBC-1 TR2 (4.2), EBC-1 TR3 (87), MKN-45 TR (17), Hs746T TR (164) |
|  | 12 | *MDM2* | Amplification | TEP-1894 (25), EBC-1 TR2 (3.5), EBC-1 TR3 (65), MKN-45 TR (6.5), Hs746T TR (104) |
| Cell line only | 1 | *NTRK1* | Amplification | EBC-1 TR2 (3.8), EBC-1 TR3 (23), MKN-45 TR (4.8) |
|  | 2 | *MYCN* | Amplification | EBC-1 TR2 (4.8), EBC-1 TR3 (138), MKN-45 TR (6.7), Hs746T TR (138) |
|  | 2 | *ALK* | Amplification | Hs746T TR2 (3.8), Hs746T Cap R (3.7) |
|  | 3 | *PIK3CA* | Amplification | EBC-1 TR2 (3.7), EBC-1 TR3 (136), MKN-45 TR (7.1), Hs746T TR (115) |
|  | 3 | *RAF1* | Amplification | EBC-1 TR3 (97), Hs746T TR (26) |
|  | 4 | *KIT, PDGFRA* | Amplification | EBC-1 TR2 (3.8), EBC-1 TR3 (139), MKN-45 TR (7.0), Hs746T TR (97) |
|  | 5 | *RICTOR* | Amplification | EBC-1 TR2 (4.5), EBC-1 TR3 (112), MKN-45 TR (7.8), Hs746T TR (282) |
|  | 7 | *BRAF* | Amplification | EBC-1 TR3 (54), Hs746T TR (125) |
|  | 7 | *EGFR* | Amplification | EBC-1 TR3 (13), MKN-45 TR (6.8), Hs746T TR (125) |
|  | 8 | *FGFR1* | Amplification | EBC-1 TR3 (72), MKN-45 TR (7.7), Hs746T TR (5.5) |
|  | 8 | *CCNE2* | Amplification | Hs746T TR2 (3.8), Hs746T Cab R (4.1) |
|  | 9 | *NOTCH1* | Amplification | EBC-1 TR2 (5.0) |
|  | 10 | *FGFR2* | Amplification | EBC-1 TR2 (4.0), EBC-1 TR3 (71), MKN-45 TR (4.2), Hs746T TR (108) |
|  | 10 | *PTEN* | Deletion | EBC-1 TR3 (0), Hs746T TR (0) |
|  | 11 | *FOLR1* | Amplification | Hs746T TR2 (3.6), Hs746T Cab R (4.6) |
|  | 12 | *CDK4* | Amplification | EBC-1 TR2 (3.5), EBC-1 TR3 (94), Hs746T TR (84) |
|  | 13 | *BRCA2* | Deletion | EBC-1 TR3 (0), MKN-45 TR (0) |
|  | 15 | *NTRK3* | Amplification | EBC-1 TR2 (4.0), EBC-1 TR3 (50), MKN-45 TR (3.8), Hs746T TR (100) |
|  | 16 | *TSC2* | Deletion | EBC-1 TR2 (0), EBC-1 TR3 (0), MKN-45 TR (0), Hs746T TR (0) |
|  | 17 | *ERBB2* | Amplification | EBC-1 TR3 (11), MKN-45 TR (4.0), Hs746T TR (73) |
|  | 17 | *RAD51D* | Deletion | EBC-1 TR3 (0.4), Hs746T TR (0) |
|  | 17 | *AURKB* | Amplification | Hs746T TR2 (3.6), Hs746T Cab R (4.0) |
|  | 17 | *TP53* | Amplification | Hs746T TR2 (3.6), Hs746T Cab R (4.0) |
|  | 19 | *CCNE1* | Amplification | EBC-1 TR2 (4.6), EBC-1 TR3 (87), MKN-45 TR (3.8), Hs746T TR (51) |
|  | 19 | *STK11* | Deletion | EBC-1 TR2 (0), EBC-1 TR3 (0), MKN-45 TR (0), Hs746T TR (0) |
|  | 20 | *AURKA* | Amplification | EBC-1 TR2 (4.4), EBC-1 TR3 (26), MKN-45 TR (4.0), Hs746T TR (124), Hs746T Cab R (4.2) |
|  | 20 | *TOP1* | Amplification | EBC-1 TR2 (4.3), EBC-1 TR3 (60), MKN-45 TR (3.6), Hs746T TR (142), Hs746T TR2 (3.7), Hs746T Cap R (3.6) |
|  | 22 | *MAPK1* | Amplification | EBC-1 TR3 (108), Hs746T TR (115) |

*^a^Greater than observed at presentation/in parental cells.
TR: tepotinib-resistant; Cab R: cabozantinib-resistant; Cap R: capmatinib-resistant; Cri R: crizotinib-resistant.*

Supplementary Table S5: **Cancer-relevant SNVs/indels detected by WES in patients.**

| **Patient** | **Gene** | **Mutation** | **Location in protein** | **CADD^a^** | **PolyPhen^b^** | **COSMIC^c^** |
| --- | --- | --- | --- | --- | --- | --- |
| TEP-153 | *ASCL1* | p.R142W | bHLH domain | 4.1 (noD) | 1.0 (PD) | 0.5% |
|  | *CDKN2A* | p.D14N | ANK1 domain | 26.5 (D) | 0.9 (PD) | 4.2% |
|  | *CSNK1A1* | p.M115T | Kinase domain | 13.7 (D) | 0.05 (B) | 0.4% |
|  | *EPHA5* | c.1927+6C>A | (Splicing) | 0.3 (noD) | -- | 7.7% |
|  | *HEY2* | p.S205L | -- | 2.1 (noD) | 0.04 (B) | 1.6% |
|  | *LIMD1* | c.14093-3_4delinsCG | (Splicing) | 0.8 (noD) | -- | 1.0% |
|  | *NKX2-1* | p.A311T | Disordered region | 2.9 (noD) | 0 (B) | 1.3% |
|  | *TBX3* | p.W197* | R2 domain | 1.0 (noD) | -- | 3.2% |
|  | *TP53* | c.993+2T>G | (Splicing) | NA | NA | 41.0% |
|  | *WEE1* | p.P131T | Regulatory domain | 1.2 (noD) | 0 (B) | 0.6% |
|  | *AQP7* | c.403+1_403+2insCAAGTG | (Splicing) | 18.7 (D) | -- | 1.5% |
|  | *ARID1A* | p.N2003Tfs*11 | GR domain | 7.2 (noD) | -- | 4.1% |
| TEP-1513 | *BCLAF1* | c.1682+1_1683-1del | (Splicing) | 22.0 (D) | -- | 7.5% |
|  | *CCN1* | p.R334W | C-terminal cystine knot domain | 2.0 (NoD) | 0.64 (PD) | 0.3% |
|  | *CTDSP2* | c.118-1G>A | (Splicing) | 0.3 (NoD) | -- | 1.0% |
|  | *DSTN* | p.T74Kfs*9 | (Frameshift) | 13.4 (D) | -- | 0.5% |
|  | *FOXD1* | c.887+1_887+2insGC | (Splicing) | 17.3 (D) | -- | 0.3% |
|  | *PCDH8* | p.G389_E394del | Disordered region | 22.9 (D) | -- | 3.7% |
|  | *RAD51B* | c.316-4_316-2delTTA | (Splicing) | 32.0 (D) | -- | 1.6% |
|  | *RYK* | c.56-1dup | (Splicing) | 11.6 (D) | -- | 0.9% |
|  | *USP14* | p.E455* | (Missense) | 38.0 (D) | -- | 0.9% |
| TEP-1894 | *AMPD1* | p.F16Y | N-terminus | 23.3 (D) | -- | 3.2% |
|  | *BCLAF1* | c.1682+1_1683-1del | (Splicing) | 22.0 (D) | -- | 7.5% |
|  | *BRCC3* | c.493-1G>T | (Splicing) | 34.0 (D) | -- | 0.9% |
|  | *CEACAM5* | p.G295* | (Nonsense) | 28.2 (D) | -- | 2.0% |
|  | *CEACAM8* | p.A164T | -- | 10.4 (D) | 0.16 (PD) | 1.1% |
|  | *FAM83B* | p.W301C | Close to the RAF1 interacting region | 27.2 (D) | 1.00 (PD)^d^ | 4.8% |
|  | *FOXD1* | c.887+1_887+2insGC | (Splicing) | 17.3 (D) | -- | 0.3% |
|  | *NOS1* | c.3042-2A>G | (Splicing) | 25.7 (D) | -- | 8.0% |
|  | *OGDHL* | p.E109* | (Nonsense) | 55.0 (D) | -- | 6.1% |
|  | *RBM10* | p.E132Rfs*67 | (Frameshift) | 22.2 (D) | -- | 6.1% |
|  | *RYK* | c.56-1dup | (Splicing) | 11.6 (D) | -- |  |
|  | *SMC1A* | p.R275L | Coiled-coil region | 23.9 (D) | 0.98 (PD) |  |
|  | *ZMYND10* | p.S364R | Close to the zinc finger | 20.4 (D) | 0.97 (PD) |  |

**^a^**D, deleterious; noD, no deleterious; using 10 as a cut-off value. In the case of small indels, the highest score of all the positions affected is shown (67).

**^b^**PD, probably damaging. PolyPhen only offers predictions for missense mutations (65).

**^c^**Frequency of mutations in lung carcinoma, according to COSMICv99 (19).

^d^Maximum score in PolyPhen.

## **Supplementary Figures**

Supplementary Figure S1

**: Spatial transcriptomic results of paired biopsies from patients’ disease progression to METi treatment. A**, Comparison of transcriptomic profile of pre-treatment and disease progressing patient samples which were treated with either tepotinib (n=2) or capmatinib (n=3). **B**, Volcano plots representing DEGs between pre-treatment and disease progressing samples from each patient. **C**, Proportion (top) of immune cells and microenvironmental cell types within each region of interest, either in the microenvironment (left) or within the tumor compartment (right). Below, scaled abundances for each segment is shown. Cell types have a maximal value of 1. Regions are clustered based on cell type composition. METi, sample, and patient for each region can be seen below the graph.

Supplementary Figure S2

**: GSEA results from individual METi post-progression patient samples.** Derived from differential gene expression of the spatial transcriptomic experiment, the GSEA results of selected hallmark terms (**A**) and C5 terms (gene ontology, **B**) from MSigDB collections are shown for each METi-progressing patient (n=5). C5 terms were selected as the GSEA output, which comprised 51 to 490 terms across patients. A p-value cut-off of 0.05 was used in all cases.

Supplementary Figure S3

**: Western blot analysis of signaling through MET, EGFR, PI3K/Akt, MAPK pathways and EMT markers. A,** Western blot of MET, PI3K/Akt, EGFR, and MAPK signaling, as well as three EMT markers, β-catenin, E-cadherin, and vimentin, were assessed in the *in vitro*-generated METi-resistant cell lines and their long-term culture control counterpart by Western blot. GAPDH or actin were used as loading controls. GAPDH, glyceraldehyde-3-phosphate dehydrogenase. TR: tepotinib-resistant; CabR: cabozantinib-resistant; CapR: capmatinib-resistant; CriR: crizotinib-resistant. **B-C**, Expression of total protein or phosphorylated protein was assessed relative to parental cells once normalization with GAPDH or tubulin was performed. This was evaluated for multiple cell lines resistant to tepotinib (**B**) and in the Hs746T cells, resistant to different METi (**C**).

Supplementary Figure S4

**: CRISPR screen quality checks. A**, Western blot indicating presence of Cas9 incorporation in the EBC-1 parental and tepotinib-resistant cells, clone 2, only induced in the presence of doxycycline. **B**, Essential gene depletion assessment as sanity checks for each CRISPR screen (C10: resistance screen in parental EBC-1 cells; C5: sensitivity screen in EBC-1 tepotinib-resistant (TR) cells) compared to the Day 0 control. **C**, PCA plot indicating differences and similarities between samples assessed. Importantly, Day 0 from both EBC-1 parental (C10) and TR (C5) cluster together, whereas a clear separation between the EBC-1 parental DMSO and tepotinib-treated cells is observed (top right). A smaller separation is also seen between the DMSO and tepotinib-treated EBC-1 TR cells (bottom right). In all cases, duplicates are in proximity.

Supplementary Figure S5

**: IC_50_ value change between EBC-1 tepotinib-resistant and parental cells for all combination partners assessed for synergy with tepotinib.** IC_50_ values for most compounds used as a combination partner to tepotinib in the synergy screen were largely unchanged between the EBC-1 parental and tepotinib-resistant cell lines, with values around 1 when a ratio was determined. METi had significantly increased IC_50_ values in the tepotinib-resistant cells, as expected, whereas the tepotinib-resistant cells were more sensitive to neratinib and dasatinib.

**References**

1.	Rosner B, Glynn RJ, Lee ML. The Wilcoxon signed rank test for paired comparisons of clustered data. Biometrics. 2006;62(1):185-92.

2.	Simon A. FastQC: a quality control tool for high throughput sequence data 2010 [Available from: <http://www.bioinformatics.babraham.ac.uk/projects/fastqc>.

3.	Chen S, Zhou Y, Chen Y, Gu J. fastp: an ultra-fast all-in-one FASTQ preprocessor. Bioinformatics. 2018;34(17):i884-i90.

4.	Harrow J, Frankish A, Gonzalez JM, Tapanari E, Diekhans M, Kokocinski F, et al. GENCODE: the reference human genome annotation for The ENCODE Project. Genome Res. 2012;22(9):1760-74.

5.	Li B, Dewey CN. RSEM: accurate transcript quantification from RNA-Seq data with or without a reference genome. BMC Bioinformatics. 2011;12:323.

6.	Ritchie ME, Phipson B, Wu D, Hu Y, Law CW, Shi W, et al. limma powers differential expression analyses for RNA-sequencing and microarray studies. Nucleic Acids Res. 2015;43(7):e47.

7.	Butler A, Hoffman P, Smibert P, Papalexi E, Satija R. Integrating single-cell transcriptomic data across different conditions, technologies, and species. Nat Biotechnol. 2018;36(5):411-20.

8.	Stuart T, Butler A, Hoffman P, Hafemeister C, Papalexi E, Mauck WM, 3rd, et al. Comprehensive Integration of Single-Cell Data. Cell. 2019;177(7):1888-902 e21.

9.	Wu T, Hu E, Xu S, Chen M, Guo P, Dai Z, et al. clusterProfiler 4.0: A universal enrichment tool for interpreting omics data. Innovation (Camb). 2021;2(3):100141.

10.	Liberzon A, Birger C, Thorvaldsdottir H, Ghandi M, Mesirov JP, Tamayo P. The Molecular Signatures Database (MSigDB) hallmark gene set collection. Cell Syst. 2015;1(6):417-25.

11.	Jolliffe IT, Cadima J. Principal component analysis: a review and recent developments. Philos Trans A Math Phys Eng Sci. 2016;374(2065):20150202.

12.	Benjamini Y, Drai D, Elmer G, Kafkafi N, Golani I. Controlling the false discovery rate in behavior genetics research. Behav Brain Res. 2001;125(1-2):279-84.

13.	NCBI. ClinVar [Available from: <https://www.ncbi.nlm.nih.gov/clinvar/>.

14.	PolyPhen-2. PolyPhen-2 prediction of functional effects of human nsSNPs [Available from: <http://genetics.bwh.harvard.edu/pph2/>.

15.	Adzhubei IA, Schmidt S, Peshkin L, Ramensky VE, Gerasimova A, Bork P, et al. A method and server for predicting damaging missense mutations. Nat Methods. 2010;7(4):248-9.

16.	University of Washington, Hudson-Alpha Institute for Biotechnology and Berlin Institute of Health at Charité. CADD - Combined Annotation Dependent Depletion [Available from: <https://cadd.gs.washington.edu/>.

17.	Schubach M, Maass T, Nazaretyan L, Roner S, Kircher M. CADD v1.7: using protein language models, regulatory CNNs and other nucleotide-level scores to improve genome-wide variant predictions. Nucleic Acids Res. 2024;52(D1):D1143-D54.

18.	Wellcome Sanger Institute. COSMIC - Catalogue of Somatic Mutations In Cancer [Available from: <https://cancer.sanger.ac.uk/cosmic>.

19.	Sondka Z, Dhir NB, Carvalho-Silva D, Jupe S, Madhumita, McLaren K, et al. COSMIC: a curated database of somatic variants and clinical data for cancer. Nucleic Acids Res. 2024;52(D1):D1210-D7.

20.	Kyoto University Bioinformatics Center. GenomeNet [Available from: <https://www.genome.jp/pathway/hsa05200>.

21.	STRING CONSORTIUM. STRING [Available from: <https://string-db.org/>.

22.	Colic M, Wang G, Zimmermann M, Mascall K, McLaughlin M, Bertolet L, et al. Identifying chemogenetic interactions from CRISPR screens with drugZ. Genome Med. 2019;11(1):52.

23.	Li W, Xu H, Xiao T, Cong L, Love MI, Zhang F, et al. MAGeCK enables robust identification of essential genes from genome-scale CRISPR/Cas9 knockout screens. Genome Biol. 2014;15(12):554.
